# Structural Origins of High MoO_3_ Solubility in Peraluminous Borosilicate Glasses

**DOI:** 10.1021/acs.jpcc.5c08536

**Published:** 2026-04-15

**Authors:** Nedgine D. I. Joseph, Hrishikesh Kamat, Rajan Saini, Grégory Tricot, Kun Wang, Randall E. Youngman, Ashutosh Goel

**Affiliations:** † Department of Materials Science and Engineering, Rutgers, The State University of New Jersey, Piscataway, New Jersey 08854-8065, United States; ‡ James R. Glidewell Dental Ceramics, Inc., Newport Beach, California 92660, United States; § Department of Physics, Akal University, Talwandi Sabo, Punjab 151302, India; ∥ Univ. Lille, CNRS, UMR 8516 – LASIRE - Laboratoire de Spectroscopie pour les Interactions, la Réactivité & l’Environnement, F-59000 Lille, France; ⊥ Kazuo Inamori School of Engineering, 1132 Alfred University, Alfred, New York 14802, United States; # Science and Technology Division, Corning Incorporated, Corning, New York 14831, United States

## Abstract

Molybdenum (Mo) imposes strict loading limits in conventional borosilicate nuclear waste glasses due to the tendency of tetrahedral molybdate [MoO_4_]^2–^ species to phase-separate and crystallize as alkali molybdates. Here, we demonstrate an unprecedented 13.96 wt % (7.51 mol %) MoO_3_ solubility in peraluminous sodium aluminoborosilicate glasses—a ∼15× increase over their peralkaline counterparts. Using Raman spectroscopy, multinuclear and dipolar-correlation magic angle spinning nuclear magnetic resonance (MAS NMR), electron paramagnetic resonance (EPR), and scanning transmission electron microscopy (STEM)-energy dispersive spectroscopy (EDS), we reveal that Na-deficient, low optical basicity conditions stabilize octahedral MoO_6_ units, which polymerize into molybdite-like Mo–O clusters dispersed within the glass matrix. These Mo-rich clusters suppress the formation of depolymerized [MoO_4_]^2–^ environments typically responsible for Na_2_MoO_4_ precipitation and instead promote the formation of Na_2_Mo_2_O_7_ as the saturation phase. Concurrently, Mo solubility drives the conversion of AlO_4_^–^ to higher-coordination AlO_5_ species, liberating Na^+^ that is subsequently sequestered in molybdate-rich domains. The combined evolution of Mo coordination, modifier redistribution, and network depolymerization provides a mechanistic basis for the markedly enhanced Mo solubility in peraluminous compositions. These findings establish new structural guidelines for designing aluminoborosilicate waste forms with substantially greater capacity to incorporate Mo-rich nuclear waste streams.

## Graphical abstract

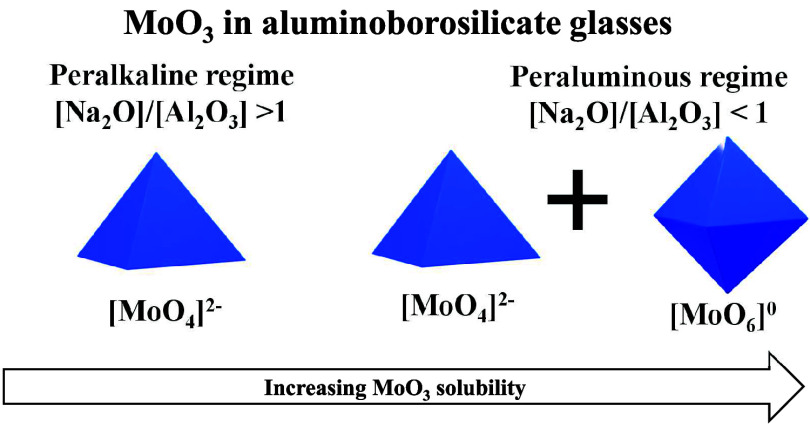

## 1 Introduction

Molybdenum (Mo) is an integral part of the high-level waste (HLW) streams produced from nuclear reactors in several countries, including the United States, France, the UK, and Japan.^1,2^ In the United States, Mo is expected to be present in the nonfissionable waste stream rich in transition metals (Zr), generated alongside streams rich in alkali/alkaline-earths (^137^Cs, ^90^Sr) and lanthanides (Ln) from the projected Transuranic Extraction (TRUEX^plus^) process.^3,4^ This process, currently under consideration by the US Department of Energy, seeks to recycle nuclear fuel by extracting fissionable materials from the spent fuel for power generation. However, the process of recycling spent fuel is projected to yield a substantial volume of secondary HLW, which is expected to be nonfissionable and must therefore be safely immobilized in a suitable waste form.

Borosilicate glasses are globally regarded as benchmark materials for immobilizing nuclear waste due to their ability to incorporate a variety of fission products within their structure.^5–7^ However, the limited solubility[Fn fn1] of molybdenum oxide (MoO_3_) into borosilicate glass (<1 mol %) severely restricts the loading capacity of the final waste form.^1,8–10^ Increasing the concentration of MoO_3_ above its solubility limit in a borosilicate glass can lead to the formation of a yellow phase comprising poorly durable alkali molybdates, e.g., Na_2_MoO_4_ and CsMoO_4_, with small concentrations of chromates and sulfates.^9,10^ The precipitation of the yellow phase can lead to premature degradation of the glassy waste form in an aqueous environment, creating pathways for the migration of certain radionuclides, such as ^137^Cs, into the geosphere. Consequently, developing glass compositions capable of accommodating higher concentrations of MoO_3_ within their vitreous matrix is a significant endeavor in the field of nuclear waste management.

Over the past few decades, extensive research has focused on elucidating the chemostructural drivers controlling the solubility of MoO_3_ in peralkaline ([M_*x*_O_*y*_]/[Al_2_O_3_] > 1; M_*x*_O_*y*_: alkali/alkaline-earth oxide) aluminoborosilicate glasses. Most of the research has been conducted on glasses melted under ambient conditions in which MoO_3_ has been reported to primarily exist as Mo^6+^, adopting a tetrahedral coordination, i.e., [MoO_4_]^2–^.^1,10^ Investigations into the local structural environment of Mo in peralkaline glasses have revealed that [MoO_4_]^2–^ units do not integrate within the aluminoborosilicate glass network but form a separate phase in the so-called depolymerized region of the glass owing to the high ionic field strength (IFS) of Mo^6+^ (IFS: 1.59–1.94 Å^–2^)­[Fn fn2],^10–15^ In the depolymerized region, the [MoO_4_]^2–^ units are charge-balanced by alkali/alkaline-earth cations present in the glass. The preferential affinity of [MoO_4_]^2–^ toward alkali/alkaline-earth cations rather than the aluminoborosilicate network promotes segregation and crystallization of alkali molybdate-based phases upon cooling of melt in steel canisters.

While most research has focused on peralkaline ([M_*x*_O_*y*_]/[Al_2_O_3_] > 1) glasses—where MoO_3_ primarily exists in 4-fold coordination, our understanding of MoO_3_ solubility in the Al_2_O_3_-rich region of alkali aluminoborosilicate glasses, i.e., the peraluminous region ([M_*x*_O_*y*_]/[Al_2_O_3_] < 1), remains limited. This compositional domain is of particular interest because, as we transition from peralkaline to peraluminous compositions, the availability of alkali/alkaline-earth cations for charge compensation of tetrahedral units such as AlO_4_^–^, BO_4_^–^, and MoO_4_^2–^ diminishes. Consequently, these structural units must compete for the limited pool of nonframework cations to maintain their tetrahedral coordination. In metaluminous ([M_*x*_O_*y*_]/[Al_2_O_3_] = 1) and peraluminous ([M_*x*_O_*y*_]/[Al_2_O_3_] < 1) glasses, B^3+^ is less effective than Al^3+^ in competing for charge compensation by alkali/alkaline-earth cations and therefore tends to adopt a 3-fold coordination, forming BO_3_ units.^16^ However, it remains unclear whether Mo^6+^ can effectively compete with Al^3+^ to retain its tetrahedral coordination. Two scenarios are conceivable as outlined below.

(1) If Mo^6+^ competes effectively with Al^3+^ for charge compensation by alkali/alkaline-earth cations, it may displace Al^3+^ from tetrahedral coordination into higher-coordination states (five- or 6-fold). Meanwhile, [MoO_4_]^2–^ units could remain in the depolymerized region of the glass until the solubility limit of MoO_3_ is reached, at which point they may phase-separate and crystallize, compromising the glass-forming ability of the system.
(2) If Mo^6+^ competes ineffectively, it may shift from tetrahedral to octahedral coordination, potentially forming MoO_6_ units within the glass matrix. Such species have been reported in highly polymerized silicate melts, including albite and rhyolite.^11^ In this case, two subscenarios can be hypothesized:
  (A) Mo^6+^, in octahedral coordination, may reduce its bond valence with oxygen from 1.5 to 1.0 valence units (v.u.), allowing MoO_6_ units to integrate into the network either by consuming residual nonbridging oxygens (NBOs) or by destabilizing bridging oxygens in the tetrahedral framework, as suggested by Farges et al.^11^ and Kim et al.^17^ in silicate melts.
  (B) Alternatively, MoO_6_ units may form clusters, leading to phase separation, as observed in phosphate glasses.^18^

In either scenario—whether through competition or lack thereof, the glass-forming ability, MoO_*x*_ solubility, and partitioning of Mo species in the glass structure are inevitably impacted.

On another note, a shift from a peralkaline to a peraluminous system reduces the optical basicity (OB) of glasses, which, in turn, can affect the redox behavior of molybdenum in glasses and melts.^19–21^ Owing to a reduced OB, a peraluminous system would have a diminished electron-donating ability (fewer O^2–^ ions), which could promote the reduction of Mo in the glass network (4M^(*z*+*n*)+^ + 2*n*O^2–^ ⇌ 4M^*z*+^ + *n*O_2_), as previously reported by Bih et al.^22^ Under these conditions, molybdenum is expected to exist as a mixture of Mo^6+^ and Mo^5+^. When discussing the impact of reduced oxidation states of molybdenum on its solubility within the aluminoborosilicate glass matrix, the literature reports conflicting findings. Some studies report that the solubility of MoO_*x*_ in silicate glasses increases with the reduction of Mo^6+^ ^20,23,24^ while others present opposing trends, suggesting decreased Mo solubility under similar conditions in silicate glasses and melts.^25–28^

These observations highlight the complex relationship among glass composition, redox conditions, and the local structural environment in governing the solubility and speciation of molybdenum in silicate-based systems. Despite substantial progress in understanding the behavior of Mo^6+^ in peralkaline glasses, critical knowledge gaps remain regarding its structural accommodation and solubility trends in peraluminous glass compositions. This gap is particularly relevant as peraluminous glasses offer promising avenues for enhancing Mo solubility by potentially altering the Mo coordination and oxidation state under lower optical basicity conditions.^15^

To address these outstanding questions, this study examines the solubility behavior and structural coordination of molybdenum in sodium aluminoborosilicate glasses formulated within the peraluminous compositional regime. The focus is on (1) the competition between MoO_4_^2–^ and AlO_4_^–^ units for Na^+^ charge compensation, (2) the potential shift in Mo coordination from tetrahedral to higher-coordination geometries under Na-limiting conditions, and (3) the influence of glass composition and structure on Mo speciation and solubility within the glass matrix. By systematically increasing the MoO_3_ content and employing a suite of complementary structural and chemical characterization techniques, this work seeks to elucidate the mechanisms governing Mo solubility in peraluminous borosilicate glasses, ultimately contributing to the design of more durable and compositionally adaptable glass waste forms for HLW immobilization.

## 2 Experimental Section

### 2.1 Glass Composition Design, Synthesis, and Compositional Analysis

The baseline glass (0Mo) has been designed in the peraluminous (Na_2_O/Al_2_O_3_ < 1) region of the sodium aluminoborosilicate system, to which increasing concentrations of MoO_3_ hasve been added to obtain a series of glasses with batched compositions: (20 Na_2_O–25 Al_2_O_3_–10 B_2_O_3_–45 SiO_2_)_(100‑*x*)_–(MoO_3_)_*x*_ (*x* = 0–10 mol %). The samples have been labeled as “*x*Mo”, where *x* is the batched concentration of MoO_3_ in mol %. The glasses were synthesized via a melt-quench technique. High-purity powders of SiO_2_ (Alfa Aesar; >99.5%), Na_2_SiO_3_ (Alfa Aesar; >99%), Al_2_O_3_ (Sigma-Aldrich; ≥98%), H_3_BO_3_ (Alfa Aesar; ≥98%), and MoO_3_ (Alfa Aesar, 99.5%) were used as precursors. Homogeneous mixtures of batches (corresponding to ∼70 g of glass) were melted in 90%Pt–10%Rh crucibles (loosely covered with a Pt lid) in the temperature range of 1500–1650 °C for 2 h in ambient atmosphere, followed by quenching of melt on a copper plate. The glasses were subsequently annealed at 0.8*T*_g_ for 2 h.^29^ The melting temperature was lowered with increasing MoO_3_ content based on the decrease in apparent viscosity of glass melts.

The resulting glass samples were crushed to fine powders with particle size <45 μm and analyzed qualitatively by X-ray diffraction (PANalytical X’Pert Pro X-ray diffractometer with Cu K_α_ radiation; 45 kV; 40 mA; 2θ range of 10–90°; step-size of 0.013°; dwell time of 0.01 s at each step) to confirm their amorphous/crystalline nature. Some samples had a salt layer adhering to their surfaces. In such cases, the salt layer was scraped off from the sample surface, followed by ultrasonication of the transparent glass sample in acetone to remove any remnant salts sticking to the sample surface. Once the salt and glass were separated, both were individually characterized via XRD. The diffraction peaks in the X-ray diffractograms of the crystalline samples were matched with those of the crystalline phases included in the International Center for Diffraction Data (ICDD) database.

The compositional analysis of the glass samples was performed using inductively coupled plasma-optical emission spectroscopy (ICP-OES; PerkinElmer ICP-OES Optima 8300 V). The methodology employed for preparing the samples for the ICP-OES analysis has been described in one of our previous articles.^30^

The glass transition temperature (*T*_g_) of the investigated glasses was determined using differential scanning calorimetry (DSC) (STA 509 Jupiter Select, Netzsch). DSC scans for powdered samples (<45 μm) were collected from 25°C to 900 °C at a heating rate of 20 °C min^–1^ under a constant flow of nitrogen gas (20 mL min^–1^). The *T*_g,onset_ values, along with the standard deviation reported in Table S6, are an average of three scans per glass composition.

### 2.2 Structural Characterization of Glasses

#### 2.2.1 Raman and Magic Angle Spinning Nuclear Magnetic Resonance (MAS NMR) Spectroscopy

The short-to-medium range ordering in the structure of the glasses has been investigated using Raman and magic angle spinning nuclear magnetic resonance (MAS NMR) spectroscopy. Raman spectra were obtained by using a Renishaw inVia Raman spectrometer under ambient conditions. A diode laser emitting at 532 nm served as the excitation source under an output power of 500 mW. The laser was directed perpendicular to a smooth sample surface, employing backscattering geometry, and focused onto the sample at a 20× magnification. The spectra were acquired within a range spanning from 200 to 1600 cm^–1^. The integration time for each sample is 100 s, with an exposure time of 10 s per cycle for 10 accumulations.

The ^29^Si MAS NMR spectra were acquired on a 9.4 T spectrometer using a 7 mm probe operating at a spinning frequency (ν_rot_) of 5 kHz, a π/2 pulse length of 5 μs, 256 transients, and a recycle delay (rd) of 300 s. The ^11^B, ^23^Na, and ^27^Al MAS NMR spectra were recorded on an 18.8 T Bruker spectrometer at 256.7, 211.6, and 208.5 MHz, respectively, using a 3.2 mm probe operating at a ν_rot_ of 20 kHz. The ^11^B MAS NMR spectra were acquired with a π/12 pulse length of 0.5 μs, 2048 transients, and a rd of 2 s. Owing to the good separation between the BO_3_ and BO_4_ overall signals, the $N_{3}\left(=\frac{BO_{3}}{BO_{3}+BO_{4}}\right)$ fraction was deduced from the relative proportions of the ^11^B fitting with satellite transition correction. The uncertainty in N_3_ corresponds to the standard deviation of the fit. The ^23^Na and ^27^Al MAS NMR spectra were obtained with a π/12 pulse length of 1 μs, 1024 transients, and a rd time of 1 s.

The ^95^Mo MAS NMR spectra were recorded at 18.8 T using a Quadrupolar Carr–Purcell–Meiboom–Gill (Q-CPMG) sequence with 40960 transients, a rd of 1 s, and spinning frequency of 10 kHz. Like many quadrupolar nuclei, ^95^Mo suffers from poor NMR sensitivity due to its low gyromagnetic ratio, low natural abundance, and strong interactions between its electric quadrupole moments and the surrounding electric field gradients.^31,32^ The Q-CPMG pulse sequence enhances the sensitivity of such nuclei by repeatedly refocusing the magnetization to generate a train of echoes. Upon Fourier transformation, these echoes yield a series of spikelets whose overall envelope reconstructs the conventional quadrupolar powder pattern.^31–33^ The ^11^B, ^29^Si, ^23^Na, ^27^Al, and ^95^Mo chemical shift values were referenced to solid NaBH_4_ (−42.06 ppm), liquid tetramethylsilane (TMS), NaCl, Al­(NO_3_)_3_, and Na_2_MoO_4_ solutions (0 ppm), respectively.

The ^27^Al­(^29^Si) dipolar heteronuclear multiple quantum coherence (D-HMQC) experiments were performed at 9.4 T using an HXY-4 mm probe operating at ν_rot_ = 8 kHz. The 2D spectra were obtained with 512 × 32 acquisition points under rotor-synchronized conditions. Each slice was recorded with ^27^Al and ^29^Si π pulse lengths of 20 and 10 μs, 2048 transients, a rd of 1 s, and a SR4_1_^2^ recoupling scheme of 2 × 1.5 ms. The ^11^B­(^29^Si) D-HMQC experiments were performed at 18.8 T on an HXY-3.2 mm probe operating at ν_rot_ = 20 kHz. The 2D spectra were obtained with 1024 × 24 acquisition points under rotor-synchronized conditions. Each slice was recorded with ^11^B and ^29^Si π pulse lengths of 20 and 8 μs, 1024 transients, a rd of 3 s, and a SR4_1_^2^ recoupling scheme of 2 × 2.0 ms. Owing to the short recoupling times used in the dipolar-mediated correlation NMR experiments, the spatial proximity revealed by our experiments could be reasonably discussed in terms of chemical connectivity.

Fitting of the obtained MAS NMR spectra was performed with DMFit.^34^ The CzSimple model^35^ was used to account for distributions in both the chemical environment and the quadrupolar coupling constant in ^23^Na and ^27^Al MAS NMR spectra. “Q MAS 1/2” and mixed Gaussian/Lorentzian peaks were used to fit the 3- and 4-fold-coordinated boron resonances in the ^11^B MAS NMR data, respectively.

#### 2.2.2 Transmission Electron Microscopy-Energy Dispersive Spectroscopy (TEM-EDS)

Thermo Fisher Scientific FEI Scios 2 DualBeam ultrahigh-resolution analytical focused ion beam, coupled with a scanning field emission electron microscope (FIB-SEM) system, was used to prepare the transmission electron microscopy (TEM) samples. The Scios 2 system operates with Ga^+^ at 1–30 keV and currents of 0.1–60 nA. The FIB is operated at 30 keV for rough milling and thinning the lamella to the desired thickness, and at 5 keV for final polishing. Scanning transmission electron microscopy (STEM) and energy dispersive spectroscopy (EDS) elemental analysis were conducted using a Thermo Fisher Scientific FEI Talos F200X operated at 200 kV. The microscope is equipped with 4 in-column super drift (Super-X) detectors for faster X-ray maps and better X-ray counts compared to conventional X-ray detectors.

#### 2.2.3 Electron Paramagnetic Resonance (EPR) Spectroscopy

The oxidation state of Mo as a function of glass chemistry was studied by using electron paramagnetic resonance (EPR) spectroscopy. Glass samples were crushed to a coarse powder using a ceramic pestle, with particle sizes ranging from 1 to 2 mm. Approximately 0.2 g of glass particles were loaded into a 5 mm borosilicate glass tube. X-band EPR spectroscopy was conducted at ambient temperature at 9.44 GHz using a Bruker EMX spectrometer and Bruker Xenon software. Continuous wave spectra were collected with a microwave power of 2.056 mW, a magnetic field modulation amplitude of 2 G, a modulation frequency of 100 kHz, and averaging over 4 scans. Double integrals of the Mo^5+^ signal, spanning 3200–3900 G, were normalized to EPR cavity quality factor (*Q*) and sample mass and then compared with the normalized EPR signal from a glass having a known concentration of Cu^2+^ to determine the Mo^5+^ concentration in these glasses. The *g*-values were calibrated with a standard (DPPH, 2,2-diphenyl-1-picrylhydrazyl, *g* = 2.0036).

## 3 Results

### 3.1 MoO_3_ Solubility in Peraluminous Glass Systems

Figure 1 presents the images of melt-quenched samples synthesized in the present study. The glasses developed a progressive amber hue as the MoO_3_ concentration increased. A similar color evolution has been previously reported for Mo-containing silicate glasses with increased MoO_3_ concentration, although those glasses were melted under reducing conditions.^24,36^ Furthermore, the surfaces of glasses containing 9 and 10 mol % MoO_3_ exhibited the precipitation of a white salt layer as shown in Figure 1. The appearance of this layer was an early indication that the concentration of MoO_3_ exceeded its solubility limit in the glass network. This observation was subsequently confirmed through XRD analysis of the salt layer, which revealed the presence of sodium molybdate (Na_2_Mo_2_O_7_) crystals (Figure S2), as discussed later in this article. Thus, in this study, the experimental MoO_3_ solubility represents the maximum concentration of MoO_3_ that can be incorporated in the glass matrix without altering its amorphous character.

**Figure 1:**
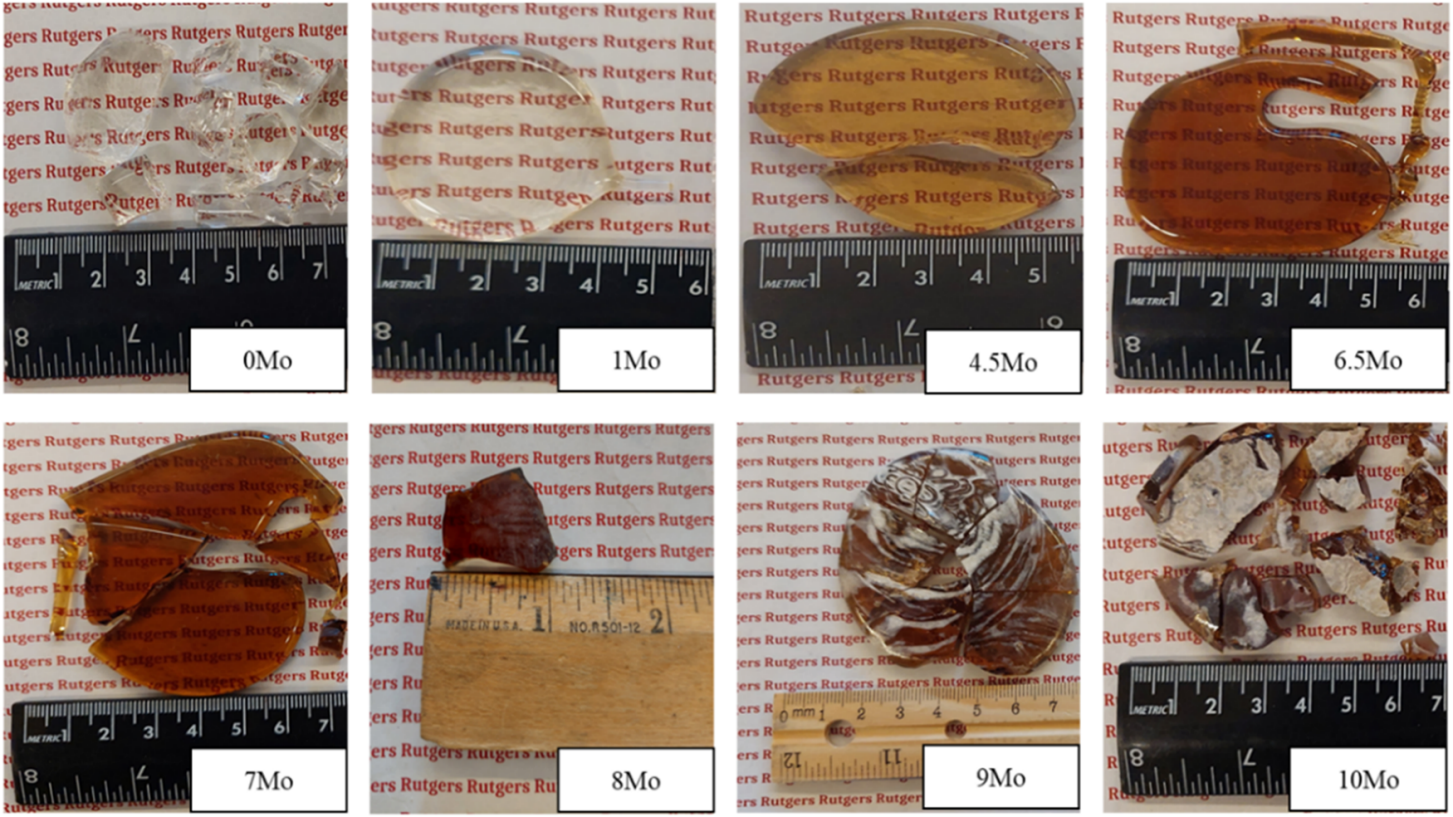
Images of the melt-quenched samples. The salt layer can be observed sticking to the surface of glasses in samples 8Mo, 9Mo, and 10Mo.

Figure S1 presents the XRD pattern of only the transparent part of the samples of the “*x*Mo” series, while Figure S2 reports the XRD pattern of the salt layer scraped from the surfaces of 9Mo and 10Mo glasses. It should be noted that a very thin layer of salt was also detected on the surface of 8Mo; however, its amount was insufficient for XRD analysis, and it was not clearly discernible in the image shown in Figure 1. All XRD patterns reported in Figure S1 confirm the amorphous nature of the glasses, whereas XRD analysis of the salt layer (9Mo and 10Mo), in Figure S2, reveals the presence of orthorhombic Na_2_Mo_2_O_7_ (PDF# 04-017-3872) as the only crystalline phase. Based on these observations, it can be inferred that the fine salt layer observed on the surface of the 8Mo sample also corresponds to the orthorhombic Na_2_Mo_2_O_7_ crystalline phase. Thus, the solubility limit of MoO_3_ in these peraluminous glasses can be defined as the amount of MoO_3_ incorporated into the glassy matrix of the 8Mo sample, as measured by ICP-OES.

Table 1 presents the experimental compositions of baseline and molybdenum-containing glasses as determined by ICP-OES analysis. Based on the data, the solubility of MoO_3_ is approximately 7.51 mol %, which represents the maximum amount that can be incorporated into the vitreous matrix of the 8Mo sample within the investigated system. This value represents a 15× increase in MoO_3_ solubility compared to a similar study on a peralkaline glass with composition 25 Na_2_O–5 Al_2_O_3_–10 B_2_O_3_–60 SiO_2_ (mol %), where MoO_3_ solubility was reported to be only 0.5 mol %.^1^ The low solubility of MoO_3_ in peralkaline alkali/alkaline-earth aluminoborosilicate glasses is, in fact, a well-documented phenomenon in the literature.^37–39^ The batched and analyzed compositions of the glasses exhibit close agreement, indicating minimal volatility of Na_2_O and B_2_O_3_ from the glass melts. Consequently, all structure–property correlations discussed in this study should be interpreted as manifestations of the impact of MoO_3_ addition to the glasses.

**Table 1:** Batched and Analyzed Compositions in mol % of the Synthesized Glasses Analyzed Using ICP-OES^a^

label		Na_2_O	Al_2_O_3_	B_2_O_3_	SiO_2_	MoO_3_
0Mo	batched	20.00	25.00	10.00	45.00	
	analyzed	18.51 (0.21)	25.73 (0.35)	9.55 (0.06)	46.20 (1.06)	0.01
1Mo	batched	19.80	24.75	9.90	44.55	1.00
	analyzed	19.32 (0.40)	25.63 (0.38)	9.77 (0.10)	44.29 (1.01)	1.00 (0.01)
4.5Mo	batched	19.10	23.88	9.55	42.98	4.50
	analyzed	18.20 (0.13)	24.22 (0.33)	9.61 (0.12)	43.71 (0.81)	4.26 (0.04)
6.5Mo	batched	18.70	23.38	9.35	42.08	6.50
	analyzed	19.11 (0.29)	23.91 (0.15)	8.89 (0.11)	42.34 (0.48)	5.76 (0.06)
7Mo	batched	18.60	23.25	9.30	41.85	7.00
	analyzed	17.26 (0.14)	23.69 (0.40)	9.32 (0.08)	43.27 (0.80)	6.46 (0.09)
8Mo	batched	18.40	23.00	9.20	41.40	8.00
	analyzed	18.66 (0.23)	23.42 (0.23)	9.54 (0.13)	40.87 (0.53)	7.51 (0.09)

a Errors associated with each component are shown in parentheses. Results are the average of six measurements (duplicate samples per composition; each measured thrice).

High-Angle Annular dark-field (HAADF)-STEM imaging was used to capture images of the 6.5Mo sample, as shown in Figure 2. This sample was selected because its MoO_3_ concentration was well below the solubility limit. TEM analysis revealed a high density of spherical, isolated clusters dispersed throughout the glassy matrix, indicative of nucleated droplet phase separation. These clusters had an average diameter of 20.7 ± 2.3 nm. Figure 2c highlights two larger particles, encircled in red, with sizes 129.7 and 193.8 nm. The amorphous nature of these clusters is confirmed by the convergent beam electron diffraction (CBED) pattern shown in Figure S3, which displays a diffuse halo devoid of discrete diffraction spots. EDS analysis was performed to investigate qualitative information about the elemental composition of these clusters. While it was challenging to acquire elemental distribution data for the fine clusters, the EDS analysis of larger clusters (indicated by the white arrow in Figure 3) confirmed that these clusters were enriched in Na and Mo, whereas Si, Al, and O were uniformly distributed in the glassy matrix. However, we acknowledge that the current EDS map does not allow a definitive determination of whether Si and Al are present within the droplets due to spatial overlap and signal mixing from the surrounding matrix. A definitive determination of droplet morphology and composition would require higher-resolution 3D analysis (e.g., atom probe tomography), which is beyond the scope of this study.

**Figure 2:**
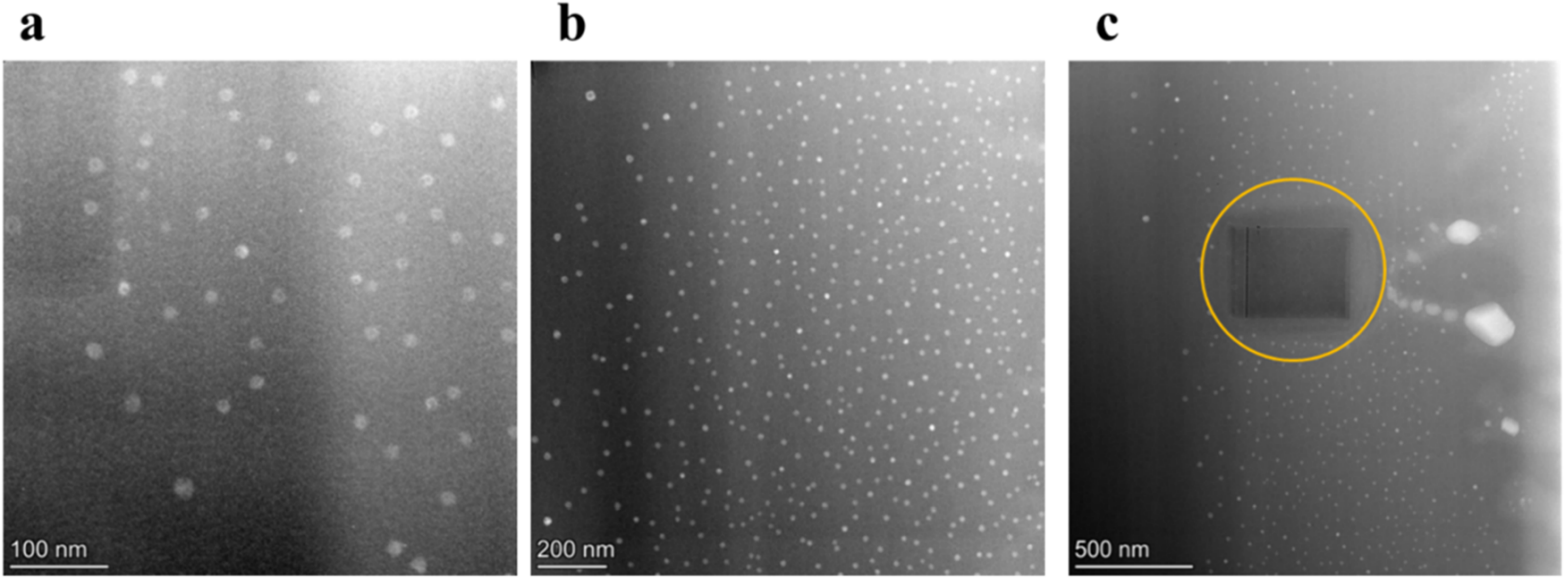
High-angle annular dark-field scanning transmission electron microscopy (HAADF-STEM) images of the 6.5Mo glass composition. Images (a, b) reveal the presence of fine clusters, while image (c) displays larger agglomerates. The yellow-circled region in image (c) corresponds to electron beam-induced damage.

**Figure 3:**
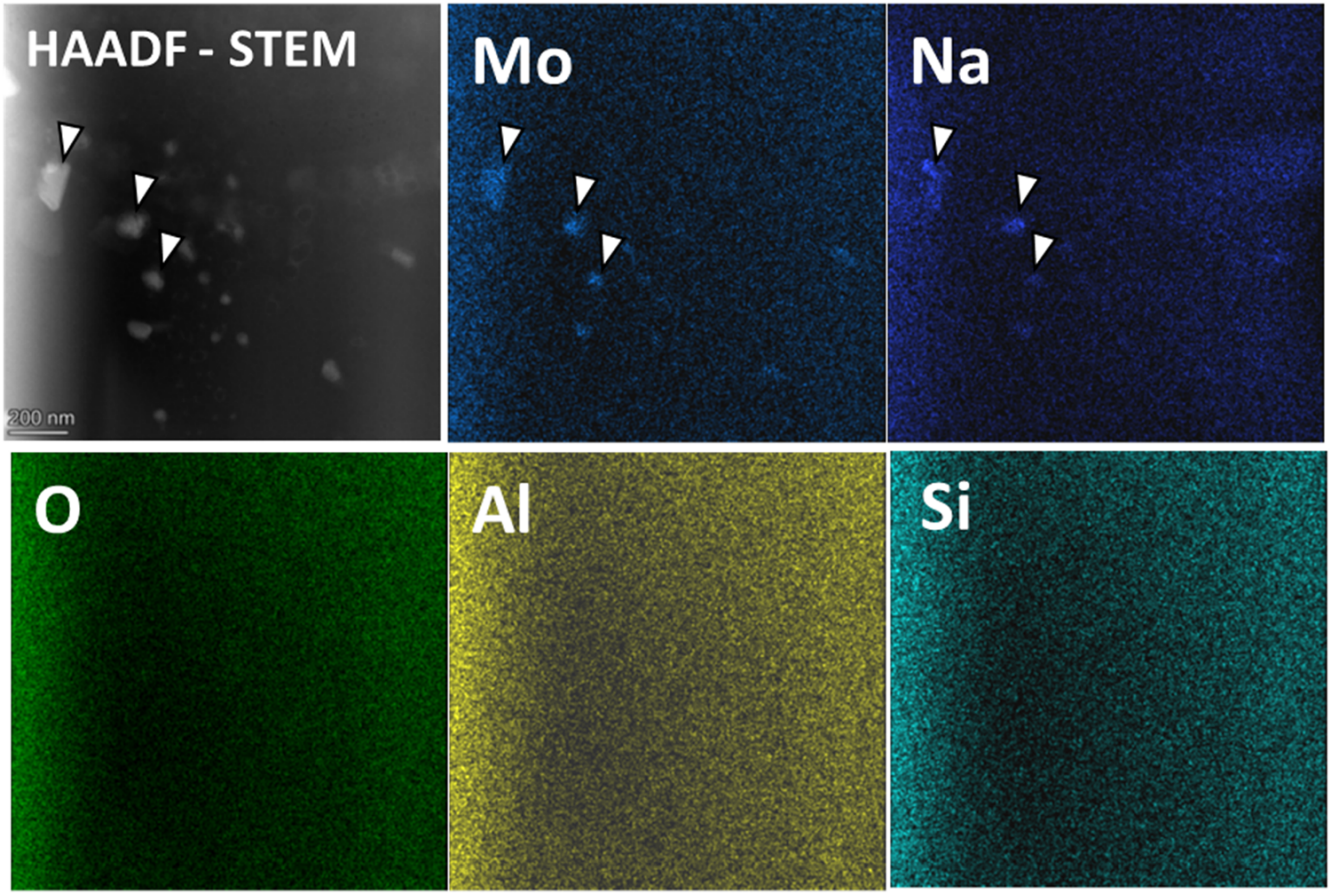
Elemental mapping of the 6.5Mo glass reveals large droplets or clusters enriched in Na and Mo. In contrast, Al, Si, and O exhibit a uniform spatial distribution across the matrix.

### 3.2 Impact of MoO_3_ on the Short-to-Medium Range Ordering in the Glass Structure

#### 3.2.1 EPR Spectroscopy

Before exploration of the structural impact of MoO_3_ addition and the underlying reasons for its high solubility in the investigated glass system, it is essential to understand the redox behavior of molybdenum within this matrix. Figure 4 presents the normalized EPR spectra of glasses 1, 4.5, and 7Mo, as a function of their *g*-values. The *g*-value of 1.924 is a characteristic of molybdenyl moieties, where molybdenum exists in the Mo^5+^ oxidation state and is coordinated by ligands (X) such as O, Cl, or S, forming [Mo^5+^=O]­X*_n_* units (where *n* = 3–6). These moieties are characterized by two types of bonds: a short, covalent, Mo=O double bond, and three–six comparatively weaker Mo–X (typically, Mo–O) covalent bonds.^11^ Analysis of the EPR spectra (Figure 4) involved the double integration of the Mo^5+^ signal to quantify the peak area. The results reveal that the concentration of Mo^5+^ in the investigated glass system ranges from 1 ppm (0.000142 wt % Mo^5+^ as Mo_2_O_5_) to 237 ppm (0.0336 wt % Mo^5+^ as Mo_2_O_5_). Thus, a 237× increase in Mo^5+^ is observed with only 7× increase in MoO_3_ concentration (from 1 to 7 mol %). Although these are small concentrations, the EPR data lack clear hyperfine coupling to Mo nuclear spins, indicating dipolar broadening and clustering of the Mo^5+^ species.

**Figure 4:**
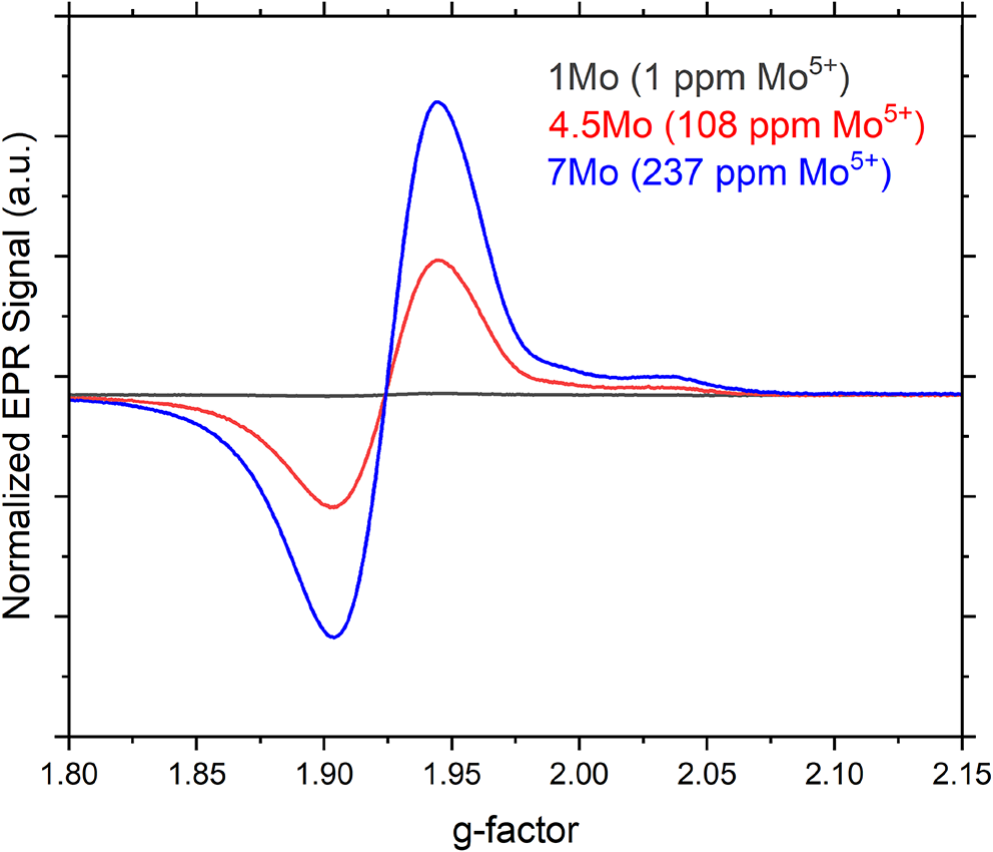
Normalized EPR spectra of glasses containing 1, 4.5, and 7 mol % MoO_3_. The progressive increase in signal intensity near *g* ≈ 1.924 with MoO_3_ content reflects a corresponding rise in Mo^5+^ concentration. Quantitative Mo^5+^ values are provided in the legend.

A comprehensive review of the literature suggests that although Mo^5+^ has been observed in 6-fold coordination—comprising one Mo=O and five Mo–O bonds—in various glass systems such as borophosphate, sodium disilicate, and sodium trisilicate glasses,^11,40^ its exact structural role remains ambiguous. For instance, Farges et al.^11^ suggested that Mo^5+^ functions as a network modifier due to its relatively high bond valence (∼0.7 v.u.), which would require coordination with a significant number of nonbridging oxygens (NBOs). However, applying the bond valence theory to classify cations as network formers or modifiers has notable inconsistencies. For example, in another study by Farges et al.,^41^ Ti–O bonds—despite having a similar bond valence (∼0.7 v.u.)—were proposed to connect with bridging oxygens in silicate melts. This was justified by the similarity in bond valence between Ti–O (∼0.7 v.u.) and Al–O (∼0.75 v.u.) bonds, suggesting the potential for Ti to form Si–O–Ti linkages within the glass network. By this logic, the six-coordinated Mo^5+^ should also participate in the silicate network rather than associating primarily with NBOs. These examples highlight the limitations of relying solely on bond valence theory to determine the structural roles of high-field-strength cations in glass networks. Therefore, further investigation is essential to clarify the local environment and structural function of Mo^5+^ in multicomponent silicate glasses. However, this will require a higher concentration of Mo^5+^ in the glass structure, which will form the basis of future studies.

#### 3.2.2 Raman Spectroscopy

Figure 5 presents the Raman spectra of the investigated glasses. In the low-frequency region (300–850 cm^–1^), the spectral bands are assigned to the mixed stretching and bending modes of T–O–T (T: tetrahedral units such as Si or Al) along with ring-breathing modes.^42–44^ In aluminoborosilicate glasses, the bands between 300 and 500 cm^–1^ correspond to the mixed stretching and bending modes of Si–O–T bonds. In Al_2_O_3_-free silicate/borosilicate glasses, the band near 488 cm^–1^ is associated with Si–O–Si bending vibrations.^43,44^ However, in aluminosilicate/aluminoborosilicate glasses, the 488 cm^–1^ band has been previously correlated to Si–O–Al vibrations, where one silicon atom is replaced by aluminum.^45,46^ The origin of Raman bands between 550 and 850 cm^–1^ in glass 0Mo is debatable. In borosilicate glasses, bands in this region are often attributed to ring-breathing modes. Specifically, the band near 575 cm^–1^ has been associated with the presence of reedmergnerite-type rings, composed of three SiO_4_ and one BO_4_ unit,^43,44,47^ while the band around 782 cm^–1^ is typically linked to vibrations of four-coordinated boron in diborate units.^44,47,48^ However, given the peraluminous nature of glass 0Mo and minimal presence of BO_4_ units in the glass structure, as deduced from ^11^B MAS NMR spectroscopy (please refer to Section 3.3.3), alternative structural contributions need to be considered. In the present study, the 575 cm^–1^ band has been assigned to the transverse motion of bridging oxygens in Al–O–Al linkages, while the 782 cm^–1^ band has been attributed to the presence of five-coordinated aluminum (AlO_5_) units and oxygen triclusters.^49^

**Figure 5:**
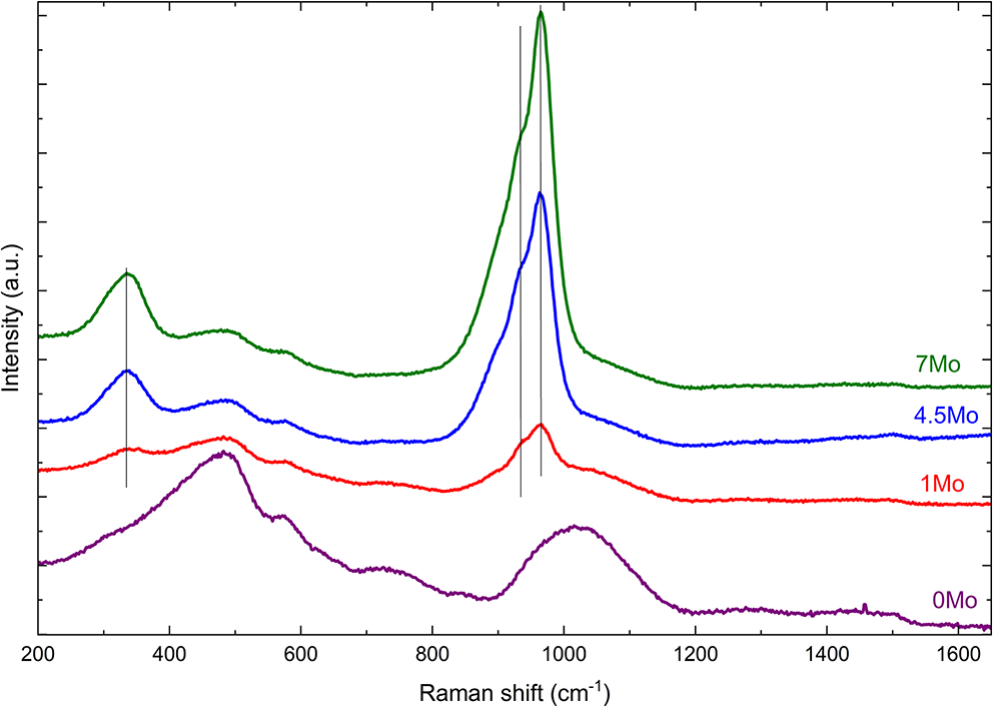
Raman spectra of sodium aluminoborosilicate glasses with varying MoO_3_ concentrations (*x* = 0, 1, 4.5, and 7 mol %).

Upon addition of MoO_3_, the spectral bands in the low-frequency region become more distinctly pronounced, most likely due to high polarizability of molybdate units. The addition of 1 mol % MoO_3_ to the baseline glass resulted in the appearance of a band at 341 cm^–1^ whose intensity increased with increasing concentration of MoO_3_. Saraiva et al.^50^ assign this band to the bending vibrations of the octahedral (MoO_6_) and tetrahedral (MoO_4_) molybdate units based on the Raman spectrum of crystalline Na_2_Mo_2_O_7_. Other authors^15,51,52^ have assigned bands between ∼320 and ∼340 cm^–1^ to tetrahedral (MoO_4_) bending vibrations.

The intermediate frequency region (850–1250 cm^–1^) is known as the silicate stretching region, with bands assigned to Si–O^–^ stretching vibrations with various *Q* ^*n*^ units (*Q* is the degree of polymerization, *n* is the number of bridging oxygens).^43,44,47,48^ With the addition of MoO_3_, two additional peaks appear in this region at 940 and 967 cm^–1^. The shoulder peak at 940 cm^–1^ has been observed in polymolybdates such as Mo_2_O_7_^2–^ and Mo_7_O_24_^6–^ and is attributed to the stretching vibration of terminal oxygen atoms in mono-oxo molybdates (Mo=O).^50,52–55^ The presence of Mo=O indicates the existence of free molybdates, which suggests that MoO_3_ is clustering within the glass matrix.^52^ Alternatively, Saraiva et al.^50^ have attributed the 940 cm^–1^ band to the stretching vibrations of oxygen atoms connecting two MoO_6_ octahedra, whereas Tsuryu et al.^56^ suggested that this band may correspond to MoO_4_ tetrahedral units and/or highly distorted MoO_6_ moieties.^53^ Furthermore, Santagneli et al.^55^ have assigned this band to terminal oxygen atoms (Mo=O or Mo–O^–^) bond vibrations associated with either tetra-, penta-, or octa-coordinated molybdenum atoms. The sharp band at 967 cm^–1^ peak corresponds to terminal Mo–O^–^ stretching vibrations within the MoO_6_ octahedra.^54,57–59^

As the MoO_3_ content rises, the characteristic spectral features of the silicate stretching region diminish in intensity due to the higher Raman scattering efficiency associated with Mo–O vibrations. Transition metals such as MoO_3_ possess a high density of d-electrons near the Fermi energy, leading to strong electron–electron and electron–phonon interactions.^60^ These interactions create phonon anomalies and phase transitions that contribute to their intense Raman scattering efficiency.^60^ At 1Mo, a leftward shift occurs in the silicate stretching region, likely due to longer bond lengths in Mo–O than Si–O bonds. According to Hardcastle and Wachs,^61^ the calculated Mo–O bond lengths for MoO_4_ and MoO_6_ units are ∼1.759 and 1.882 Å, respectively, while the bond length for the mono-oxo species is ∼1.681(16) Å. In contrast, the Si–O bond length is approximately 1.63 Å.^62^

### 3.3 Solid-State Nuclear Magnetic Resonance Spectroscopy

#### 3.3.1 Sodium Environment in Glasses

The ^23^Na MAS NMR spectra of the glasses display a single, broad, asymmetrical peak, with the quadrupolar coupling constant (*C*_Q_) remaining relatively constant at 1.1 MHz, indicating no significant broadening with increasing MoO_3_ content (Figure S4). However, the peak maxima shift toward lower isotropic chemical shift (δ_iso_) from −9.9 to −11.4 ppm with MoO_3_ addition. This upfield shift suggests a change in the Na^+^ environment—potentially reflecting an increase in the Na–O bond distance or a transition from Na^+^ associating primarily with AlO_4_^–^ units to other structural units.^43,46,63–66^ As supported by TEM analysis (Figure 3), Na^+^, in addition to charge-compensating AlO_4_^–^ units, are also aggregated within the molybdate-rich, phase-separated region of the glass matrix. These clusters likely require Na^+^ ions for charge compensation, reinforcing the shift in the Na^+^ environment from aluminate-to molybdate-based units.

#### 3.3.2 Aluminum Environment in Glasses

In peraluminous glasses, the concentration of Al^3+^ exceeds the concentration of modifier cations. Therefore, all Al^3+^ cannot be adequately charge-balanced in four-coordination. Thus, to maintain charge neutrality, two structural models have been proposed: (1) excess Al^3+^ transitions to higher-coordination states (AlO_5_ and AlO_6_ units), and/or (2) formation of oxygen triclusters where an oxygen atom interlinks with three tetrahedral moieties.^67–72^ The first structural behavior is reflected in the ^27^Al MAS NMR spectra of all of the glasses. As MoO_3_ content increases from 0 to 7 mol %, the fraction of AlO_5_ increases from 7 ± 2% to 13 ± 2% (Table S2). This increase in aluminum coordination can be attributed to the higher ionic field strength (IFS: 1.59–1.94 Å^–2^) of Mo^6+^. Due to its high IFS, Mo^6+^ competes with AlO_4_ (hereafter also referred to as ^[4]^Al) for available oxygen in order to lower its coordination number, thus promoting the formation of higher-coordinated aluminum species, as illustrated in eq 1 ^73^

1 A[4]l+M[n]⇌A[5]l+M[n−1]

where ^[*n*]^M represents a high ionic field strength cation (M) with coordination number *n*.

Since the majority of Al^3+^ in the investigated glasses is four-coordinated, it is reasonable to assume that Mo^6+^ could not effectively compete with Al^3+^ for oxygen. However, owing to its higher IFS compared to another network modifying cation (e.g., Na^+^), Mo^6+^ could still influence the oxygen environment around Al^3+^. This effect is evident from the broadening in the ^27^Al MAS NMR spectra of glasses with increasing MoO_3_ concentration, as reflected by the rising *C*_Q_ values (5.5–5.8 MHz) shown in Table S2.

There is a slight decrease in the AlO_4_ isotropic chemical shift (δ_iso_) from 64.1 to 63.3 ppm with increasing MoO_3_ addition. This shift can be attributed to (i) the substitution of more electronegative cations (e.g., Mo^6+^), (ii) an increase in angle(s) between tetrahedra (Al–O–T), (iii) a decrease in the average Al–O distance,^71,74,75^ (iv) variations in the SiO_2_/Al_2_O_3_ ratio, or (v) demixing of Al and Si. However, there is an insignificant change in the SiO_2_/Al_2_O_3_ ratio in the glass compositions (Table 1), so the contribution from (iv) is expected to be minor. The isotropic chemical shifts for the AlO_5_ units were ∼40 ppm, and the *C*_Q_ values ranged from 2.8 to 4.6 MHz. As *C*_Q_ reflects the degree of symmetry of Al polyhedra, higher *C*_Q_ values indicate a more distorted AlO_5_ environment.^46^

Figure 6a presents the ^27^Al–^29^Si 2D correlation maps of glasses with MoO_3_ content varying between 0 and 7 mol % along with the normalized ^27^Al and ^29^Si projections on the horizontal and vertical axes, respectively. The corresponding nonnormalized ^27^Al and ^29^Si projections are shown in Figure 6b accompanied by the simulations. As summarized in Table 2, these nonnormalized projection intensities are compared together using the highest-intensity projection in the series as a 100% reference. The overall associated error was evaluated on a single sample with two different 2D experiments performed with an identical experimental procedure. The comparison aims to evaluate the extent of Al/Si mixing within the glass network. On this basis, a comparison of ^27^Al and ^29^Si projections suggests a slight decrease in Al/Si mixing at higher MoO_3_ contents (4.5 and 7 mol %), with the largest reduction observed for the 7Mo sample. Generally, the addition of high IFS cations leads to a decreased adherence to Loewenstein’s rule, promoting more Al–O–Al linkages at the expense of Al–O–Si linkages, thereby reducing Al/Si intermixing.^71,76–78^ It should be noted that the extent of Al/Si linkages—and consequently the intensity observed in the 2D map projections—is composition-dependent. While the molar ratio of Al_2_O_3_-to-SiO_2_ remains mostly constant across all the compositions, their relative concentrations diminish with increasing MoO_3_ content. This compositional shift possibly leads to a reduction in Al/Si mixing, even under otherwise constant structural conditions. Nonetheless, this compositional trend alone is unlikely to fully account for the observed decrease in the intensity of 2D map projections.

**Figure 6:**
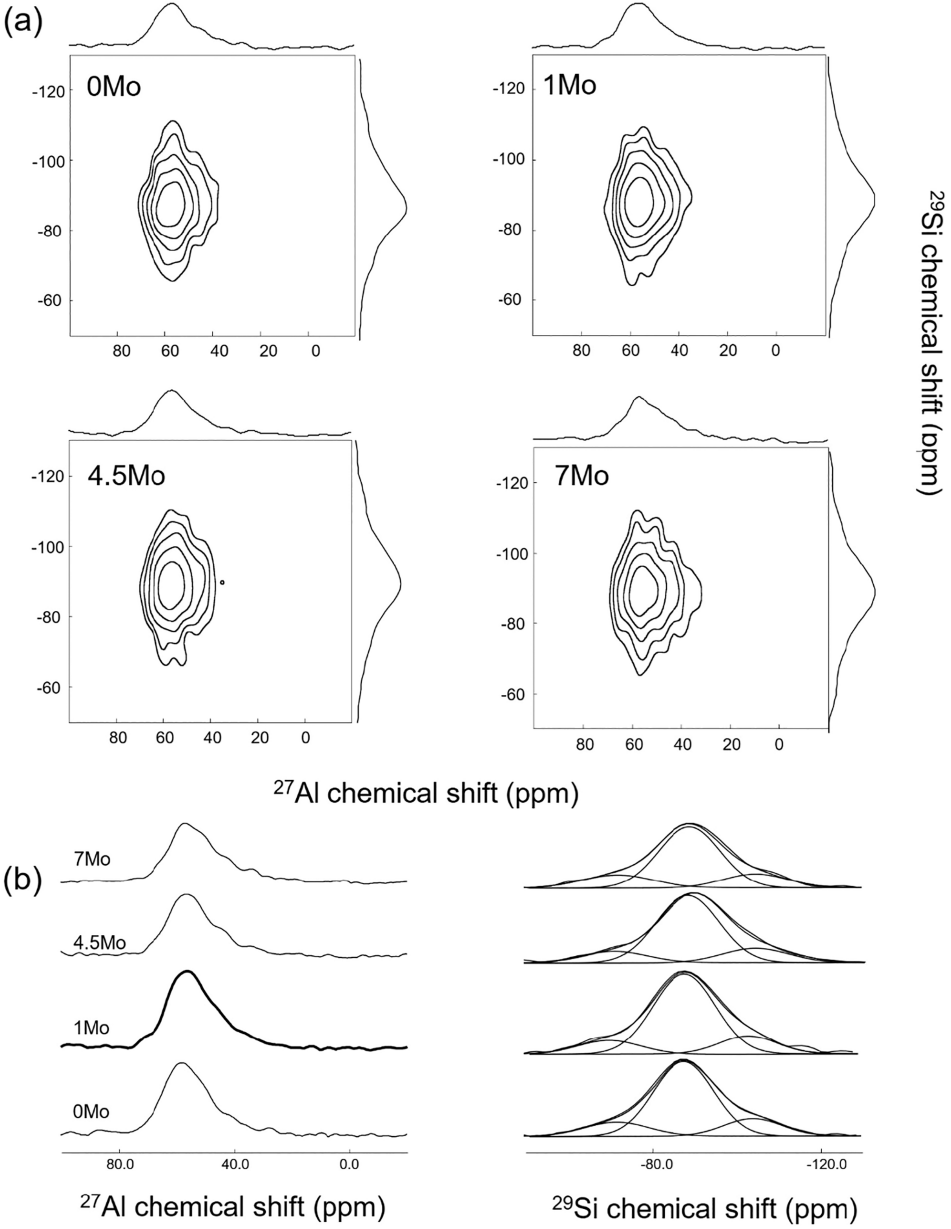
(a) 2D ^27^Al/^29^Si correlation maps along with the normalized ^27^Al and ^29^Si projections in the horizontal and vertical axes and (b) ^27^Al and ^29^Si nonnormalized 2D map projections.

**Table 2:** Comparison between the Nonnormalized 2D Map Projections Magnitude^a^

	Al/Si projection intensities		B/Si projection intensities	
sample	^27^Al	^29^Si	^11^B	^29^Si
0Mo	99%	100%	92%	97%
1Mo	100%	100%	100%	100%
4.5Mo	91%	100%	80%	82%
7Mo	82%	88%	80%	79%

a The percentages are given with an error of ±5%.

In addition to this semiquantitative information presented above, the simulations of the ^29^Si projection spectra (Figure 6b) reveal the presence of three distinct silicate species associated with Al-containing units. The corresponding resonances, centered at −71, −87.5, and −103 ppm, are observed in all the investigated compositions, albeit with varying relative intensities. In conventional alkali silicate glasses, signals in this chemical shift range are typically assigned to *Q* ^1^, *Q* ^2^, and *Q* ^3^ units, chemical shift values are influenced not only by the degree of silicate polymerization but also by the number of neighboring Al atoms. Consequently, the structural environments are more appropriately described by using extended *Q^n^*_*m*Al_ notation, where *n* and *m* represent the number of connected Si and Al tetrahedra, respectively. Unambiguous assignment of specific *Q^n^*_*m*Al_ species is therefore challenging due to overlapping contributions arising from the combined effects of *n* and *m*. For instance, the resonance at approximately −87.5 ppm could be assigned to *Q*_4Al_^0^ or *Q*_3Al_^1^ based on a previous correlation study on ^29^Si-enriched calcium aluminosilicate glasses.^79^ Since such isotopic enrichment and correlation NMR experiments were not feasible for the present samples, further quantitative analysis of silicate speciation was not possible. Nevertheless, a more precise description of the silicate network polymerization would be valuable for a deeper understanding of Mo partitioning in these glasses.

#### 3.3.3 Borate Environment in Glasses

The ^11^B MAS NMR spectra for all investigated glasses displays a dominant broad resonance centered between 10 and 20 ppm, attributed to trigonal borate (BO_3_) units, alongside a smaller peak near 0 ppm corresponding to tetrahedral borate (BO_4_) species. In the peraluminous baseline glass (0Mo), Na^+^ ions are expected to preferentially charge-compensate AlO_4_^–^ units rather than BO_4_^–^, thereby favoring the formation of BO_3_ units.^80,81^ This hypothesis is strongly supported by the MAS NMR data, as the fraction of trigonal borate units, i.e., $N_{3}\left(=\frac{BO_{3}}{BO_{3}+BO_{4}}\right)$ , exceeds 99% in the 0Mo glass. Moreover, the incorporation of MoO_3_ has a minimal effect on borate speciation across the glass series. Detailed fitting parameters for the BO_3_ units are provided in Table S3. These deconvolutions have been performed using the widely used ring/nonring models.^46,82–84^

Figure 7a displays the ^11^B/^29^Si 2D correlation maps, along with the ^11^B and ^29^Si normalized projections in the horizontal and vertical axes, respectively. The correlation maps exhibit a strong signal across all glasses, indicating significant interactions between the borate and silicate networks, primarily as ^[3]^B–O–Si linkages. The degree of B/Si mixing is expressed through the nonnormalized 2D map projections reported in Figure 7b and their comparison (Table 2). As already observed for the Al/Si mixing, introduction of MoO_3_ contents higher than 4.5 mol % leads to a decrease of the B/Si mixing. This change correlates with a slight increase in the fraction of ring BO_3_ units (from 7 ± 1% in 0Mo to 11 ± 1% in 7Mo). According to Du and Stebbins,^82^ ring BO_3_ species preferentially connect to the borate network, whereas nonring BO_3_ tend to undergo random mixing in borosilicates. The increase in ring BO_3_ species likely promotes ^[3]^B–O–^[3]^B over ^[3]^B–O–Si linkages, thereby decreasing B/Si intermixing with increasing MoO_3_ content. The ^29^Si projections have been deconvoluted using a single-component model (Figure 7b), showing that only one *Q^n^*_*m*B_ species is present in our glasses independent of the MoO_3_ content. The signal appears at −83 ppm, but as previously discussed for the *Q^n^*_*m*Al_ species, no assignment can be unambiguously proposed.

**Figure 7:**
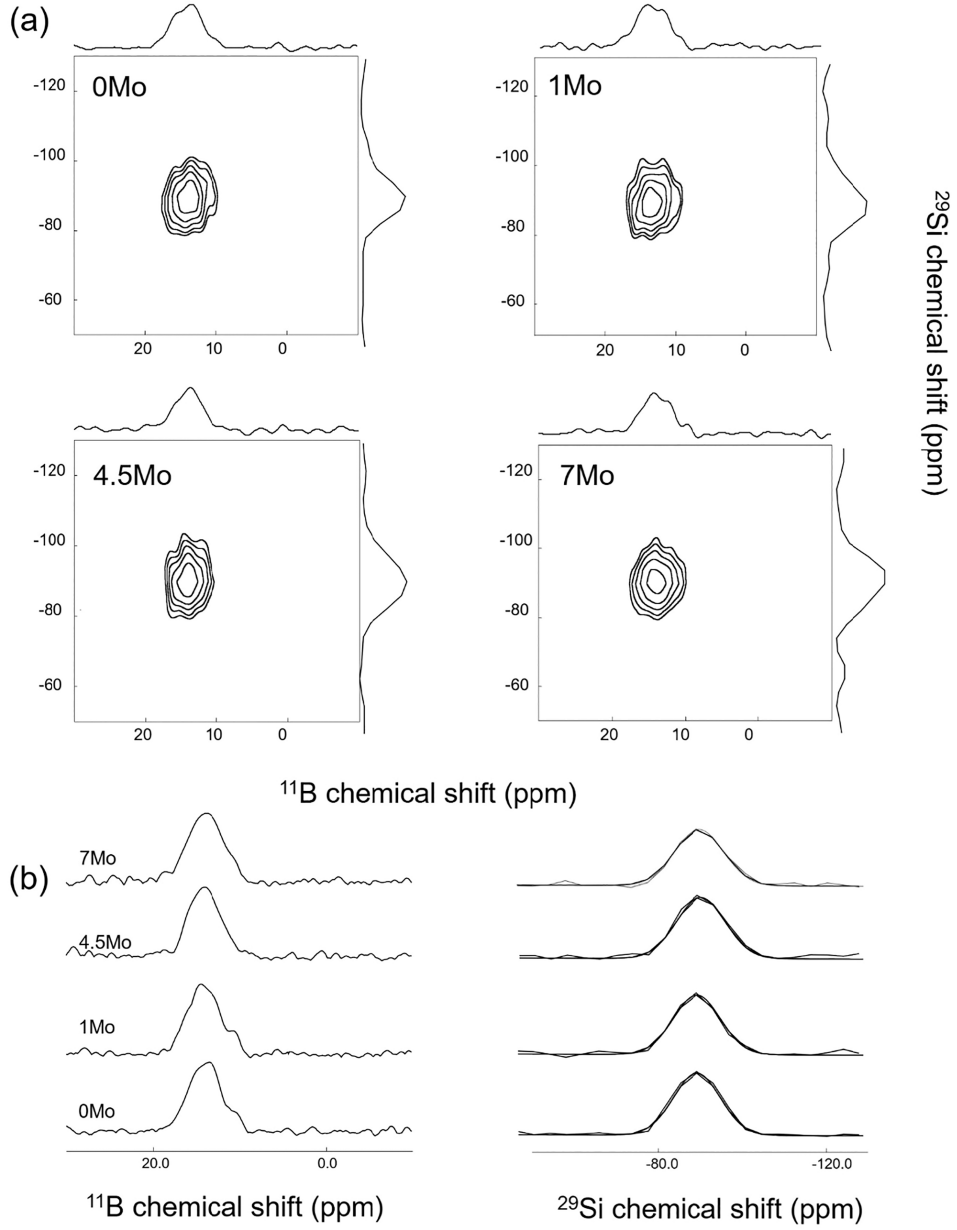
(a) 2D ^11^B/^29^Si correlation maps along with the normalized ^11^B and ^29^Si projections in the horizontal and vertical axes and (b) ^11^B and ^29^Si nonnormalized 2D map projections.

#### 3.3.4 Molybdenum Environment in Glasses

Figure 8 presents the ^95^Mo Q-CPMG NMR spectra of the investigated glasses. ^95^Mo is a quadrupolar nuclei (*I* = 5/2) with low natural abundance (15.72%) and a relatively low Larmor frequency (52.3 MHz at 18.8 T).^85,86^ ^95^Mo Q-CPMG at 18.8 T collected on crystalline Na_2_Mo_2_O_7_ (salt layer precipitated on the glass surface) resolves two sites: [4]Mo (δ_iso_ = −26 ppm; *C*_Q_ = 1.2 MHz) and octahedral ^[6]^Mo (δ_iso_ = −15 ppm; *C*_Q_ = 4.1 MHz). In the crystalline compound, the octahedral site shows a less negative δ_iso_ and markedly larger *C*_Q_ than do the tetrahedral sites. The *C*_Q_ values for both sites are consistent with those reported for crystalline alkali orthomolybdates (CaMoO_4_ and Na_2_MoO_4_) and pyromolybdates (Na_2_Mo_2_O_7_ and K_2_Mo_2_O_7_).^87^ The spectra indicate that MoO_4_ species exhibit relatively narrow resonances, suggesting a more symmetric structural environment.^86^ In contrast, the spectra of the MoO_6_ species show considerable second-order quadrupolar broadening, which accounts for the larger *C*_Q_ value of MoO_6_ compared to MoO_4_, as detailed in Table S4.

**Figure 8:**
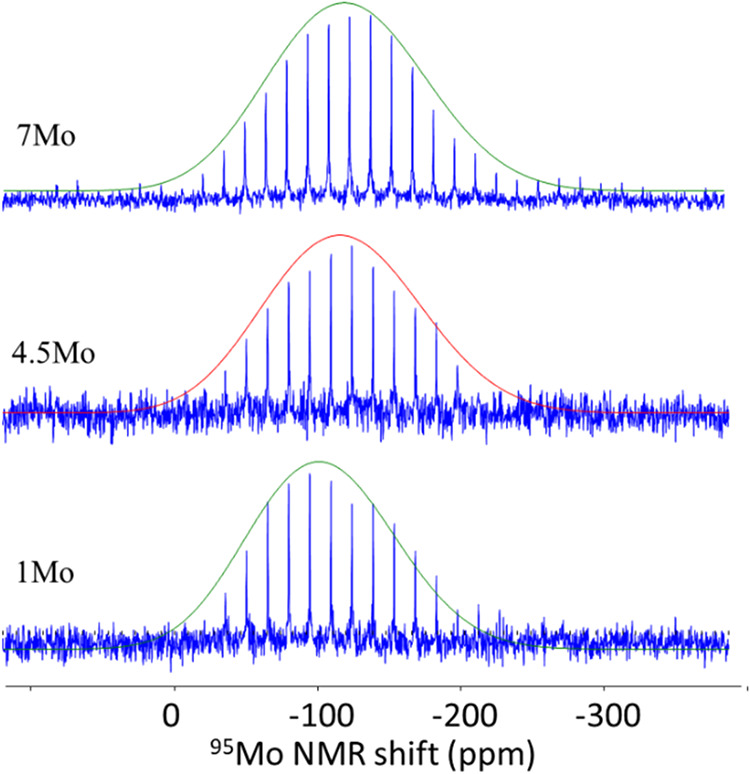
^95^Mo Q-CPMG NMR spectra of *x*Mo (*x* = 0–7) glasses.

In the glasses investigated in this study, broad signals centered near −100 ppm are observed across representative compositions. With increasing MoO_3_ content, the peak position shifts slightly to lower frequencies and becomes broader. It is noteworthy that the observed range is broader than that observed for crystalline Na_2_Mo_2_O_7_, where δ_iso_ values for ^[4]^Mo and ^[6]^Mo are −26 and −15 ppm, respectively.

In the literature, the assignment of molybdenum coordination in glasses is somewhat ambiguous. In the NaPO_3_–MoO_3_ glass system, the resonances near 0 and −500 ppm in ^95^Mo MAS NMR spectra were tentatively assigned to ^[4]^Mo and ^[6]^Mo, respectively.^55^ In borophosphate systems, broad resonances centered near −100 ppm are assigned to ^[4]^Mo.^40^ Similarly, in alkali borosilicates, the broad resonance near 0 to −100 ppm is assigned to ^[4]^Mo.^88,89^ In a study conducted by Shi et al.,^90^ the broad resonance centered around +50 ppm has been assigned to ^[4]^Mo, while the one near −60 ppm has been assigned to ^[6]^Mo in aluminoborosilicate glasses. Therefore, caution should be exercised when interpreting ^95^Mo MAS NMR spectra to study the coordination environment of molybdenum in glasses. Although the coordination environment of molybdenum from ^95^Mo MAS NMR spectroscopy is inconclusive, the Raman spectra in Figure 5 does suggest the coexistence of both tetrahedral and octahedral units.

## 4 Discussion

### 4.1 Impact of MoO_3_ on the Structure of Peraluminous Glasses

#### 4.1.1 Aluminum Environment

In peraluminous glasses, the available Na^+^ ions are insufficient to fully charge-compensate the AlO_4_^–^ units, leading a portion of Al^3+^ to adopt higher-coordination states (AlO_5_). Assuming that Mo^6+^ cannot effectively compete with Al^3+^ for Na^+^ charge compensation and thus cannot maintain tetrahedral coordination, any Mo^6+^ present in the glass would be expected to exist primarily in octahedral coordination, as MoO_6_ units are electrically neutral. However, upon addition of MoO_3_ to the studied glass system, the fraction of AlO_5_ units increases steadily. This trend suggests that Mo^6+^, due to its higher IFS, competes with AlO_4_^–^ for the available oxygen atoms. As a result, the coordination number of Mo^6+^ decreases (from ^[6]^Mo → ^[4]^Mo), while the population of higher-coordinated aluminum species increases, as illustrated in eq 1. Since ^[5]^Al units are electrically neutral,[Fn fn3],^91^ their formation releases Na^+^ ions, which are subsequently utilized to charge-compensate [Mo_2_O_7_]^2–^ (= [MoO_4_]^2–^ + [MoO_3_]­[Fn fn4]) units within the glass network. Therefore, Mo^6+^ does not necessarily compete directly with Al^3+^ for Na^+^; instead, it induces structural rearrangements that liberate Na^+^ for molybdate charge compensation. The AlO_4_ → AlO_5_ transition and concomitant Na^+^ ions release are supported by the presence of Na–Mo-rich clusters observed in TEM-EDS analyses, and by the upfield shift in the ^23^Na MAS NMR spectra, reflecting the redistribution of Na^+^ ions from aluminate to molybdate environments. Further, increasing the MoO_3_ content reduces Al/Si intermixing, suggesting decreased adherence to Loewenstein’s rule and the progressive replacement of Al–O–Si linkages with Al–O–Al and Si–O–Si linkages. The influence of high ionic field strength cations, for example, Ca^2+^, La^3+^, Y^3+^, on the aluminate environment in alumino­(boro)­silicate glasses has been well-documented in the literature.^1,73,92,93^

#### 4.1.2 Borate Environment

BO_3_ units (>99%) are the predominant borate species in this study, due to the dearth of Na^+^ ions needed for charge-compensating BO_4_^–^ units. With an increasing MoO_3_ content, there is a slight increase in the fraction of BO_3_ ring units, which could explain the decrease in Si/B intermixing in the glass network. Considering that borate units preferentially associate with the silicate network through BO_4_ units first, followed by BO_3_ nonring, and then BO_3_ ring units, a reduction in nonring BO_3_ would be expected to lower the overall connectivity between the silicate and borate networks.^82^ Similar decreases in Si/B mixing have been reported in alkali alumino- and borosilicates.^88,94,95^ However, Caurant et al.^96^ and Brehault et al.^1^ reported that the BO_4_ population was not significantly impacted by the MoO_3_ content. Thus, the minor changes observed in boron speciation may be influenced by the glass chemistry.

#### 4.1.3 Molybdenum Environment

In an alkaline aqueous solution, molybdenum predominantly exists as MoO_4_^2–^. As the pH decreases, MoO_4_^2–^ undergoes hydrolysis and subsequent polymerization to polyanions such as heptamolybdate [Mo_7_O_24_]^6–^ and octamolybdate [Mo_8_O_26_]^4–^, in which MoO_6_ octahedra are linked by sharing edges and corners.^97–101^ During hydrolysis, protonation of the oxygen ligands (O^2–^ → –OH) lowers their electron-donating ability and reduces their per-bond valence contribution to Mo^6+^. The donor strength of the coordinating ligands (O^2–^) strongly influences the metal’s (Mo^6+^) coordination: complexes with weaker electron-donating ligands tend to adopt higher-coordination numbers (MoO_6_), whereas those with stronger electron-donating power form stronger bonds and can stabilize lower coordination ([MoO_4_]^2–^).^102,103^

Although glass is not an aqueous solution, the concept of optical basicity treats it as a solution of oxide components (SiO_2_, Na_2_O, Al_2_O_3_, B_2_O_3_, and MoO_3_) in an oxide-ion network. Therefore, the acidity or basicity of glass can be expressed in terms of optical basicity—the electron-donating power of oxide anions.^104^ Low optical basicity corresponds to a more weakly electron-donating oxide environment, analogous to an increased acidity in an aqueous system. Under such conditions, the reduced donor strength of O^2–^ lowers the average bond valence per Mo–O bonds, favoring the distribution of bond valence over a large number of bonds and thereby stabilizing higher coordination of Mo^6+^.

In peraluminous systems, the low Na_2_O–to–Al_2_O_3_ ratio lowers the optical basicity and drives the formation of octahedral molybdate units, as corroborated by Raman spectroscopy. Similarly, in metaluminous systems ([Na_2_O]/[Al_2_O_3_] = 1), as reported by Farges et al.,^11^ minor fractions of octahedral Mo^6+^ are also observed. In peralkaline compositions, a stronger oxide electron donation stabilizes tetrahedral MoO_4_^2–^. Other transition metals (e.g., Mn^2+^, Co^2+^, Ni^2+^) exhibit a similar behavior: low-basicity glasses stabilize their octahedral coordination, whereas high-basicity glasses favor tetrahedral coordination.^105^ Section 4.2 further explores how the peraluminous nature of the glass relates to the molybdenum coordination reflected in the precipitated crystalline Na_2_Mo_2_O_7_ phase.

When correlating the optical basicity of glass with the molybdenum’s redox behavior, the incorporation of alkali and alkaline-earth cations accompanies an increase in the negative charge on the oxide ion, thereby enhancing their electron-donating ability and raising the glass’s optical basicity (i.e., creating a more alkaline environment).^19,21,106,107^ A glass with higher optical basicity requires stronger acidic species for charge neutrality; consequently, the higher oxidation state of Mo, i.e., Mo^6+^, is favored due to its greater charge density and stronger acidic character relative to Mo^5+^.^106^ Conversely, substituting these alkaline modifiers with more acidic oxides, such as phosphates or aluminates, reduces the optical basicity of the glass, thereby favoring a more reduced species of molybdenum, as made evident by Mo^5+^ in the EPR spectra.

### 4.2 Formation of Na_2_Mo_2_O_7_ Salt Phase

Within the structure of crystalline Na_2_Mo_2_O_7_, molybdenum exhibits two coordination environments comprising MoO_4_ (tetrahedral) and MoO_6_ (octahedral) units. This structural configuration is defined by the formation of infinite chains, wherein MoO_6_ octahedra share vertices within the chains, while MoO_4_ tetrahedra serve as bridges linking adjacent MoO_6_ units, as seen in Figure 9.^108,109^ According to the phase diagram of the Na–Mo–O system (Figure 10),^110^ Na_2_Mo_2_O_7_ is stable at lower Na/Mo ratios, e.g., in peraluminous systems, while Na_2_MoO_4_ becomes the favored phase at higher Na/Mo ratios, e.g., in peralkaline systems.^1,110,111^ Therefore, the formation of Na_2_Mo_2_O_7_ as the salt phase in the present investigation is justified as there is a dearth of Na_2_O considering its peraluminous nature, where the majority of Na^+^ ions are charge-compensating AlO_4_^–^, as shown using ^27^Al MAS NMR spectroscopy. Thus, the effective Na/Mo ratio in this study should be less than 1. Similar results have been reported in the alkali (A_2_O = Na_2_O, K_2_O) monomolybdates (A_2_O–MoO_3_), i.e., A/Mo > 1, where characteristic vibrations of [MoO_4_]^2–^ units are observed.^51,112^ However, when the A/Mo ratio decreases, as in dimolybdates (A_2_O–2MoO_3_), pyromolybdate [Mo_2_O_7_]^2–^ anions are formed.^51,112^ A further decrease in the A/Mo ratio could lead to the formation of tripolymolybdate [Mo_3_O_10_]^2–^ and tetrapolymolybdate [Mo_4_O_13_]^2–^ anions.^112^

**Figure 9:**
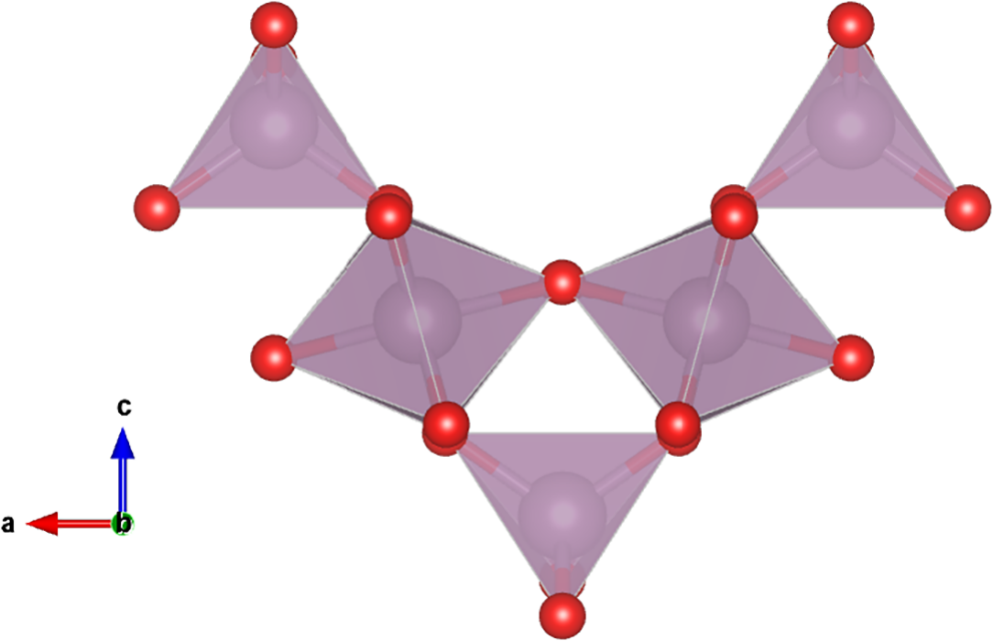
MoO_4_ tetrahedra and MoO_6_ octahedra, viewed along the *b*-axis (ac plane), were visualized by VESTA.^113^ Purple and red spheres represent molybdenum and oxygen atoms, respectively.

**Figure 10:**
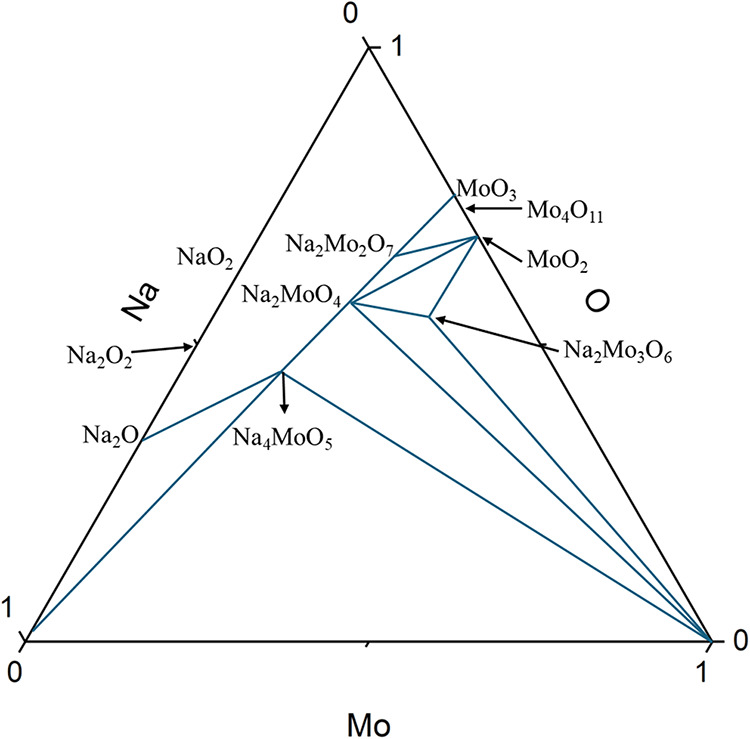
Reconstructed phase diagram of Na–Mo–O system identified by the temperature range of 673–923 K. Reprinted from Journal of Nuclear Materials, Vol. 165, Gnanasekaran, T.; Mahendran, K. H.; Kutty, K. V. G.; Matthews, C. K., Phase diagram studies on the Na–Mo–O system, 210–216, Copyright (1989), with permission from Elsevier.

Considering the abovementioned rationale for the formation of the Na_2_Mo_2_O_7_ salt phase in peraluminous glasses due to the dearth of Na^+^ resulting in Na/Mo < 1, one should also expect a similar behavior in metaluminous glasses since the ratio of Na_2_O–to–Al_2_O_3_ in these glasses is also equal to 1, i.e., the majority of Na^+^ ions are used for charge-compensating AlO_4_^–^ units, as has been shown in our previous studies.^16,114^ Therefore, the effective Na/Mo ratio in these glasses should also be low, similar to the peraluminous glasses. In such a scenario, the solubility of MoO_3_ and the chemistry of salt phase (upon saturation of MoO_*x*_ in the glass melt) in metaluminous ([Na_2_O]/[Al_2_O_3_] = 1) glasses should be similar to their peraluminous ([Na_2_O]/[Al_2_O_3_] < 1) analogues. Thus, to test the validity of this hypothesis, another series of glasses with the composition 25Na_2_O–25Al_2_O_3_–10B_2_O_3_–40SiO_2 (100–*x*)_–(MoO_3_)_*x*_, where [Na_2_O]/[Al_2_O_3_] = 1, was designed and synthesized. Nonetheless, the results on metaluminous glasses reveal a significantly lower MoO_3_ solubility (∼1.55 mol %) in the glassy matrix compared to their peraluminous analogues. At 2.5 mol % MoO_3_ in the batched composition of metaluminous glass, a salt phase was observed on the glass surface, which upon XRD analysis (Figure S7) revealed the formation of a mixture of Na_2_MoO_4_ (PDF #04-010-8839) and Na_2_Mo_2_O_7_ (PDF #04-017-3872) crystalline phases.

In contrast, in the peraluminous glass discussed here, only Na_2_Mo_2_O_7_ precipitated, with no evidence of Na_2_MoO_4_ formation. This suggests a shift in the molybdenum environment within the glass toward more polymerized molybdate complexes, specifically Mo_2_O_7_^2–^, where molybdenum atoms form chains composed of both MoO_4_ and MoO_6_ polyhedrons. This polymerization reduces the amount of sodium required for charge compensation of the molybdate complexes. Based on the results observed in the present investigation, we propose the following chemical equation (eq 2) to describe the shift toward Na_2_Mo_2_O_7_, analogous to the equation outlined by Machida and Eckert^86^

2 $$Na_{2}\mathrm{Mo}O_{4}+\mathrm{Mo}O_{3}↔Na_{2}Mo_{2}O_{7}$$

In the metaluminous melt, the conversion of Na_2_MoO_4_ to Na_2_Mo_2_O_7_ is incomplete. However, in the peraluminous melt, the higher MoO_3_ content forces the remaining MoO_4_^2–^ moieties to further polymerize into Mo_2_O_7_^2–^ complexes. This behavior is consistent with the Na_2_MoO_4_–MoO_3_ binary phase diagram,^115,116^ as depicted in Figure S9. At low MoO_3_/Na_2_MoO_4_ ratios, Na_2_MoO_4_ is the more stable phase, while higher ratios favor the stability of Na_2_Mo_2_O_7_.

### 4.3 Structural Origins of High MoO_3_ Solubility

Under Na^+^-deficient conditions, Mo^6+^ is unable to maintain its tetrahedral [MoO_4_]^2–^ configuration and starts to transition into an octahedral MoO_6_ coordination within the glass matrix. Based on this observation, two hypotheses are proposed in Section 1 to describe the structural role of MoO_6_ units.

#### 4.3.1 Network Integration via Reduced Bond Valence

According to the bond valence model, the sum of bond valences around an oxygen atom must closely approximate the theoretical value of 2.0 valence units (2.0 ± 0.1 v.u.). It is theoretically feasible for octahedrally coordinated Mo^6+^ to connect to the tetrahedral network, as the bond valence sum for ^[4]^Si–O–^[6]^Mo, ^[3]^B–O–^[6]^Mo, and ^[4]^Al–O–^[6]^Mo linkages would be 2.0, 2.0, and 1.75 v.u., respectively.^11,117^ Despite this possibility, there has not been any clear experimental evidence in the literature confirming the presence of ^[6]^Mo–O–(Al, B, Si) linkages. However, prior work by Tricot et al.^40^ has proposed the possibility of ^[4]^B–O–Mo linkages supported by the emergence of a new borate species at high MoO_3_ concentrations in borophosphate glasses. Additionally, Kim et al.^17^ have attributed an improved MoO_3_ solubility in aluminoborosilicate glass matrices to the possible formation of ^[6]^Mo^6+^–O–Si and/or Mo^5+^/Mo^4+^–O–Si linkages.

#### 4.3.2 Clustering and Phase Separation

TEM analysis of 6.5Mo revealed amorphous nanoscale, nucleated droplet-like phase separation. These clusters were enriched in Na and Mo, suggesting that some Na^+^ ions are charge-compensating molybdate complexes at the expense of [AlO_4_]^−^ units, as is evident by the shift of Al^3+^ to higher coordination with increasing MoO_3_ content. This is consistent with the behavior of other highly charged cations (e.g., W^6+^, Cr^6+^), which tend to form isolated clusters in glass networks and strip modifiers from network formers to stabilize their own domains.^118^

While the authors do not rule out the possibility of ^[6]^Mo–O–(Al, B, Si) linkages in the investigated glasses, the present study shows clear evidence of the clustering and phase separation of molybdenum complexes within the glass matrix. This validates hypothesis B as the main structural role of octahedral Mo^6+^ in the peraluminous glass matrix.

As the Mo content increased, a small fraction of Mo^6+^ was reduced to Mo^5+^; however, the role of Mo^5+^ remains unclear in the literature. Given that Mo^5+^ forms only in trace amounts (<1 wt %), its influence on the glass structure is assumed to be minimal. Thus, the gradual change in coordination of Mo^6+^ from four to six is proposed as the primary factor influencing Mo solubility in the investigated glasses.

## 5 Conclusions

In this study, we examined the structural origins of high MoO_3_ solubility in peraluminous sodium borosilicate glasses by using a comprehensive suite of characterization techniques. Our findings reveal a 7.51 mol % MoO_3_ solubility in peraluminous glasses, a value significantly higher than the solubility observed in the peralkaline regime (0.5 mol %). Based on our previous studies and the results from the current investigation, the MoO_3_ solubility in alkali aluminoborosilicate glasses has been found to follow the following trend: peralkaline < metaluminous < peraluminous. Using a suite of characterization techniques, we show: (1) the formation of reduced Mo^5+^ (<1 wt %) species in the glass network, (2) increasing MoO_3_ decreases B/Si and Al/Si intermixing, and (3) a progressive shift from tetrahedral [MoO_4_]^2–^ units toward octahedral MoO_6_ species, subsequently shifting precipitation from Na_2_MoO_4_ to Na_2_Mo_2_O_7_. These molybdate complexes form clusters in the glass network and strip Na^+^ from AlO_4_^–^ units to stabilize their own domains, driving the conversion of AlO_4_^–^ to higher-coordinated AlO_5_ species. Given the low concentration of Mo^5+^ in the glass network, we attribute the enhanced MoO_3_ solubility in the investigated series to the gradual transition of Mo^6+^ from a tetrahedral to an octahedral coordination.

## Supplementary Material



## Footnotes

1 In the present work, solubility refers to the concentration of Mo that can be incorporated into the glassy matrix while maintaining an amorphous structure under an established equilibrium between dissolved and atmospheric Mo species.

2 The ionic field strength (IFS) of Mo^6+^ was calculated using the Shannon ionic radii for ions.^14^ Calculations were performed for coordination numbers ranging from 4 to 6.

3 According to the literature,^93^ AlO_5_ units are linked to tricoordinated oxygen atoms (^[3]^O) for charge compensation. The population of these ^[3]^O is primarily found in OAl_3_ and OAl_2_Si configurations.

4 MoO_3_ units here represent the MoO_6_ octahedral units in the glass structure. The structure of crystalline MoO_3_ consists of layers of distorted MoO_6_ octahedra sharing edges and corners, forming a two-dimensional network parallel to the (010) plane. These layers are held together by van der Waals forces.

## Notes

Experimental details, NMR fitting parameters, XRD diffractograms, and other materials (PDF)
