# Supplementary material for: Structural Origins of High MoO3 Solubility in Peraluminous Borosilicate Glasses
Source: J Phys Chem C Nanomater Interfaces. 2026 Apr 15;130(16):5949–64. doi: 10.1021/acs.jpcc.5c08536 (PMC13112348; doi:10.1021/acs.jpcc.5c08536)
Supplement: Supplementary file 1 [file jp5c08536_si_001.pdf]

Supporting Information

Structural origins of high MoO_3 solubility in peraluminous borosilicate glasses

Nedgine D.I. Joseph,[1] Hrishikesh Kamat,[2] Rajan Saini,[1,3] Grégory Tricot,[4] Kun Wang,[5] Randall E. Youngman,[6] Ashutosh Goel[1,*]

[1] Department of Materials Science and Engineering, Rutgers, The State University of New Jersey, Piscataway, NJ 08854-8065, United States.

[2] James R. Glidewell Dental Ceramics, Inc., Newport Beach, CA 92660, United States.

[3] Department of Physics, Akal University, Talwandi Sabo, Punjab, 151302, India.

[4] Université de Lille, CNRS, UMR 8516 - LASIR - Laboratoire de Spectrochimie Infrarouge et Raman, F-59000 Lille, France.

[5] Kazuo Inamori School of Engineering, Alfred University, Alfred, NY 14802, United States.

[6] Science and Technology Division, Corning Incorporated, Corning, NY 14831, United States.

[*] Corresponding author:
Email: ag1179@soe.rutgers.edu; Ph: +1-848-445-4512

Supporting Information

Table S1. Fitting parameters obtained for ^{23}Na MAS NMR spectra.

Sample	δ_{iso} (ppm) (± 0.2)	FWHM CS (ppm) (± 0.2)	C_Q (MHz) (± 0.1)
0Mo	-9.9	17.4	1.1
1Mo	-10.1	17.4	1.1
4.5Mo	-10.9	17.1	1.1
7Mo	-11.4	16.5	1.1

*δ_{iso} is the isotropic chemical shift, FWHM CS is the full width half maximum of the gaussian distribution of the isotropic chemical shift, and C_Q is the quadrupolar coupling constant.

Supporting Information

Table S2. NMR fitting parameters for the ^{27}Al MAS spectra.

Sample		δ_{iso} (ppm) (± 0.2)	FWHM CS (ppm) (± 0.2)	C_Q (MHz) (± 0.1)	f (%) (± 2%)	Average Al coordination (ACN)
0Mo	Al (IV)	64.1	11	5.5	93	4.07
	Al (V)	38.1	22	2.8	7	
1Mo	Al (IV)	63.9	12	5.5	91	4.09
	Al (V)	40.1	23	4.0	9	
4.5Mo	Al (IV)	63.7	12	5.8	90.	4.10
	Al (V)	37.2	23	3.0	10	
7Mo	Al (IV)	63.3	12	5.8	87	4.13
	Al (V)	39.3	25	4.6	13	

*δ_{iso} is the isotropic chemical shift, FWHM CS is the full width half maximum of the gaussian distribution of the isotropic chemical shift, f is the relative fraction of Al (IV) and Al (V), and C_Q is the quadrupolar coupling constant.

Table S3. NMR fitting parameters for the ^{11}B MAS spectra.

Sample		δ_{iso} (ppm) (± 0.2)	C_Q (MHz) (± 0.1)	etaQ (± 0.1)	f (%) (± 1%)
0Mo	B(III) ring	18.5	2.9	0.20	7
	B(III) nonring	16.9	2.7	0.20	93
1Mo	B(III) ring	18.3	2.8	0.20	8
	B(III) nonring	16.8	2.7	0.20	92
4.5Mo	B(III) ring	18.1	2.7	0.20	9
	B(III) nonring	16.6	2.7	0.20	91
7Mo	B(III) ring	17.9	2.7	0.20	11
	B(III) nonring	16.5	2.6	0.20	89

*δ_{iso} is the isotropic chemical shift, C_Q is the quadrupolar coupling constant, etaQ is the asymmetry parameter, and f is the relative fraction of B(III) ring and nonring.

Supporting Information

Table S4. Q-CPMG ^{95}Mo NMR parameters of $Na_2Mo_2O_7$. There does not appear to be a relationship between d_{CS} and C_Q of $Na_2Mo_2O_7$ and the glasses.

**This study**	**δ_{iso}(ppm) / C_Q (MHz)**	
	$^{[4]}Mo$	$^{[6]}Mo$
9.4 T (20 h)	-26 / 1.2	-15 / 4.1
18.8 T (16 h)	-25 / 1.2	-14 / 4.0

*δ_{iso} is the is the isotropic chemical shift, and C_Q is the quadrupolar coupling constant.

Supporting Information

Table S5. Batched and analyzed compositions in mol.% of the synthesized metaluminous glass with 2.5 mol.% MoO_3 analyzed using ICP-OES. Errors associated with each component are shown in parenthesis. The ICP-OES are the average of six measurements (duplicate samples per composition; each measured thrice).

Label		Na_2O	Al_2O_3	B_2O_3	SiO_2	MoO_3
Met 2.5Mo	Batched	24.38	24.98	9.75	39.00	2.50
	Analyzed	22.96	26.24	9.36	39.88	1.55
		0.67	0.33	0.06	0.59	0.02

Table S6. $T_{g,onset}$ values (mean ± standard deviation) for the investigated glasses, calculated from three scans per composition.

Label	$T_{g,onset}$ ± °C
0Mo	674.2 ± 2.8
1Mo	663.4 ± 2.4
4.5Mo	611.0 ± 2.8
7Mo	593.4 ± 2.9

Supporting Information

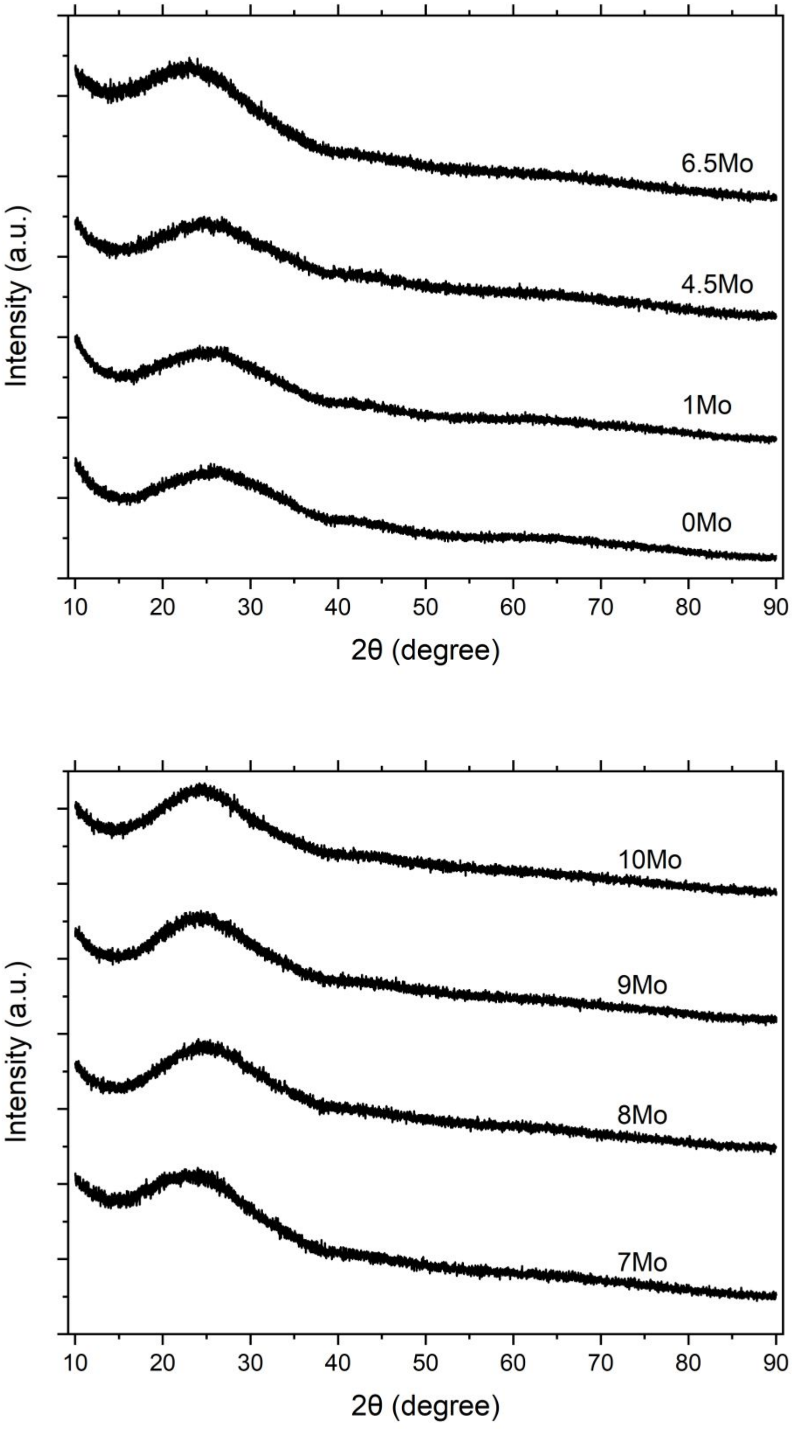

Figure S1. X-ray diffractograms of transparent matrix of molybdenum-containing glasses.

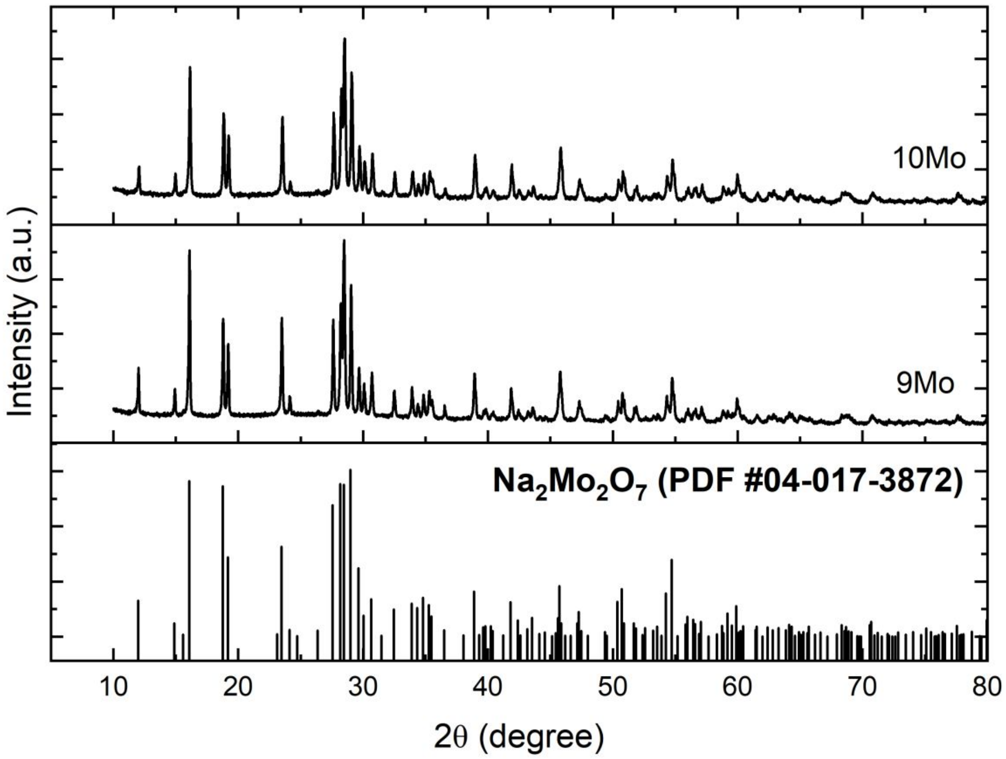

Figure S2. X-ray diffraction patterns of salt layer obtained on the surface of Mo9 and Mo10. The crystalline phase is $Na_2Mo_2O_7$ (PDF #04-017-3872).

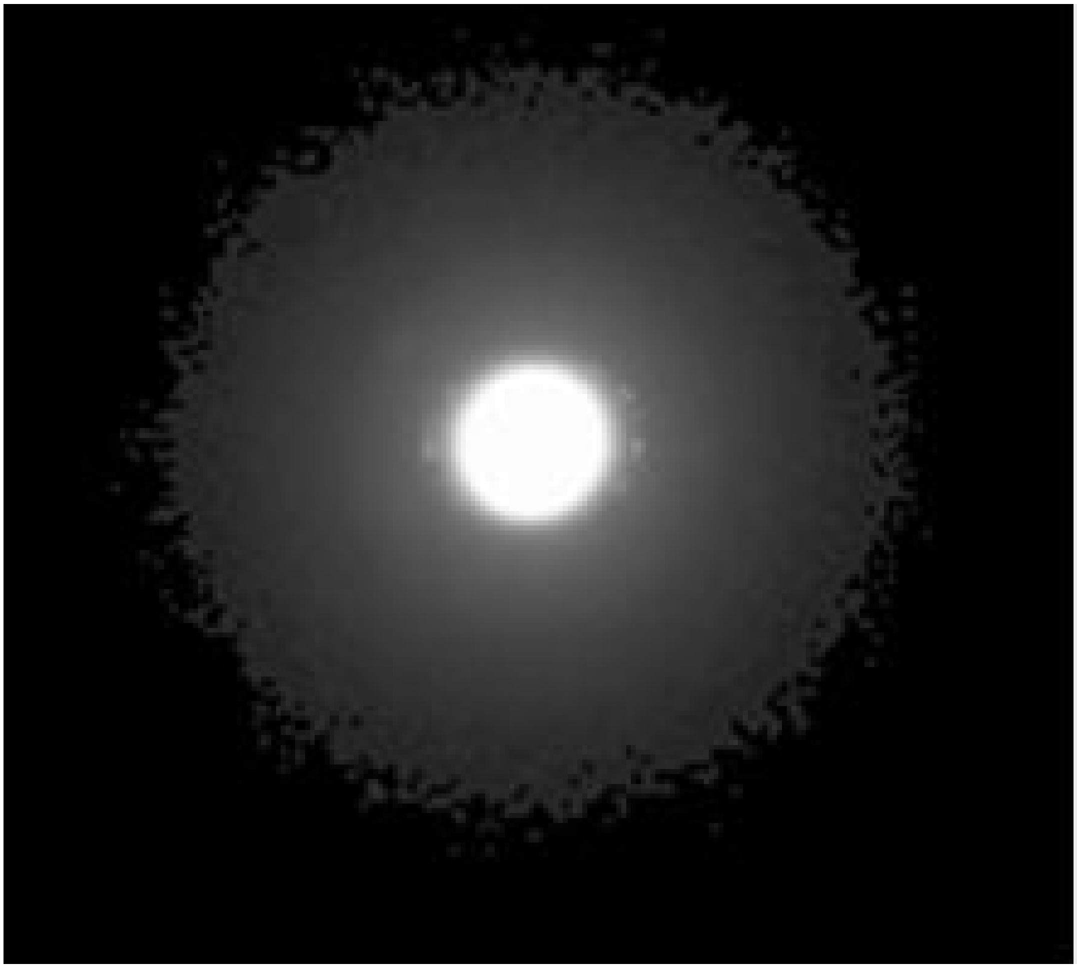

Figure S3. Convergent beam electron diffraction (CBED) under STEM mode of 6.5Mo.

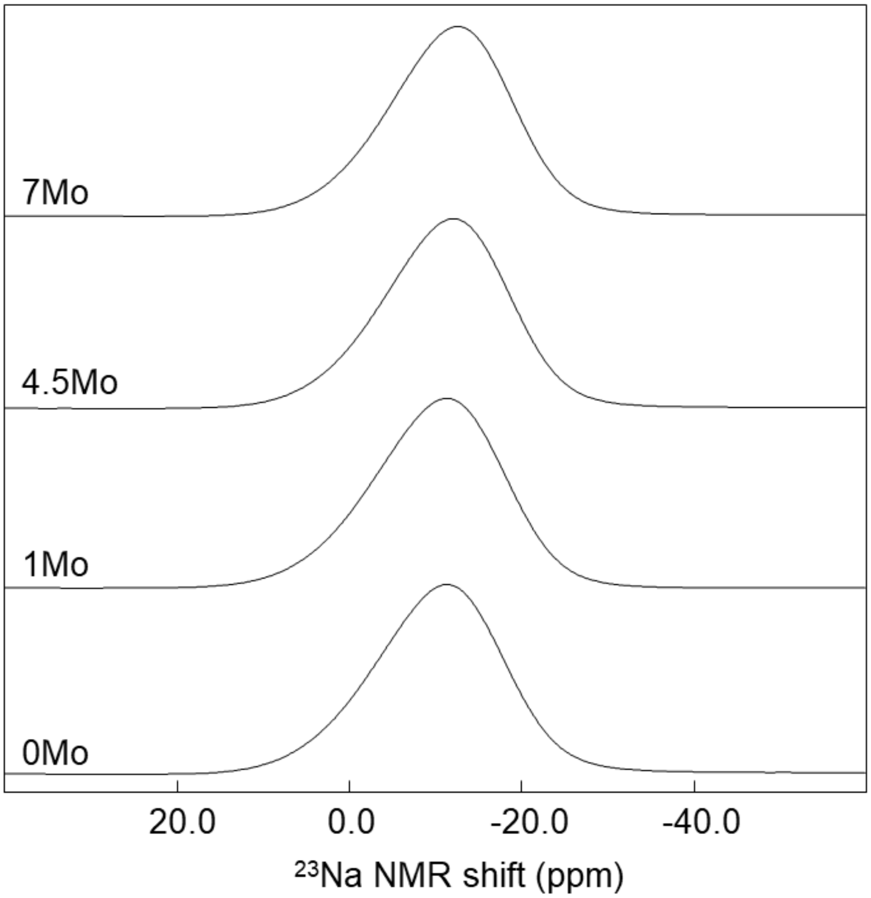

Figure S4. ^{23}Na MAS NMR spectra of xMo (x=0-7) glasses.

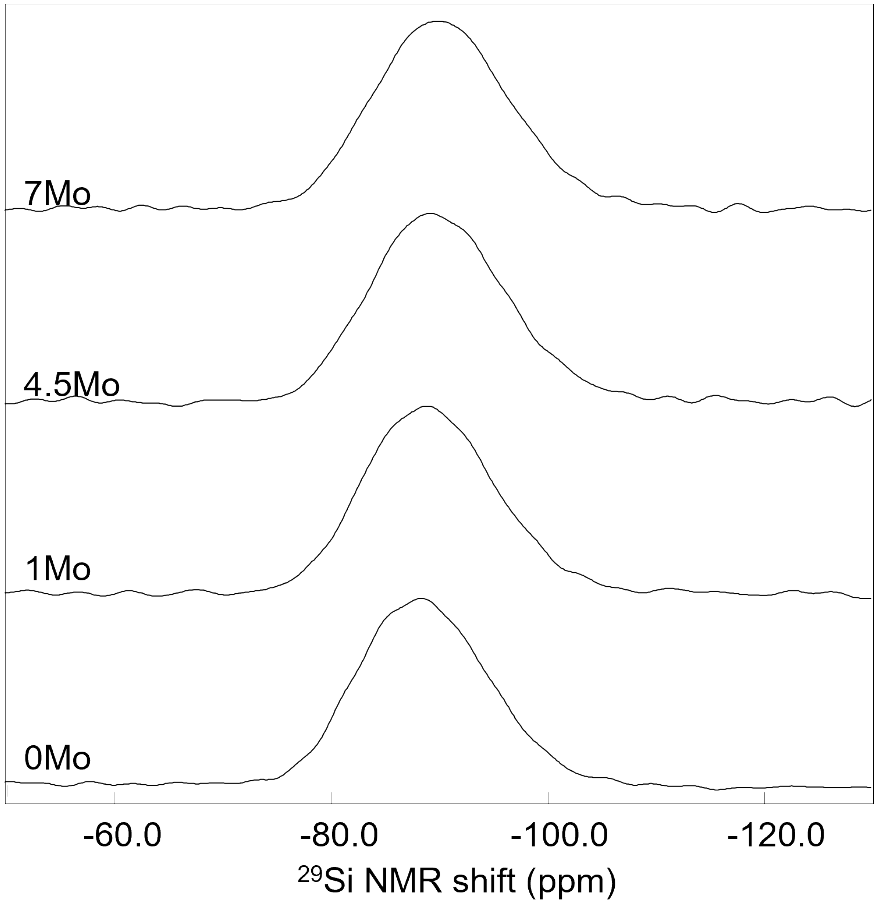

Figure S5. ^{29}Si MAS NMR spectra of xMo (x=0-7) glasses.

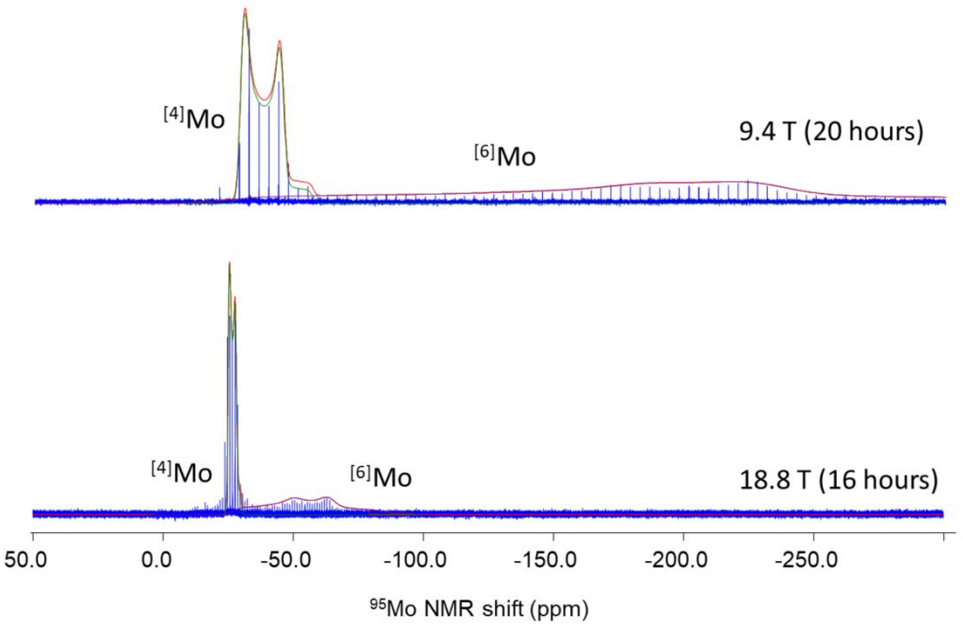

Figure S6. ^{95}Mo Q-CPMG NMR spectra of $Na_2Mo_2O_7$ at 9.4 T (20 hours) and 18.8 T (16 hours). The spectra indicate that MoO_4 species exhibit narrow resonances, suggesting a more symmetric structural environment. [1] In contrast, the spectra of the MoO_6 species show considerable second order quadrupolar broadening, which accounts for the larger C_Q value of MoO_6 compared to MoO_4 as detailed in **Table S4.**

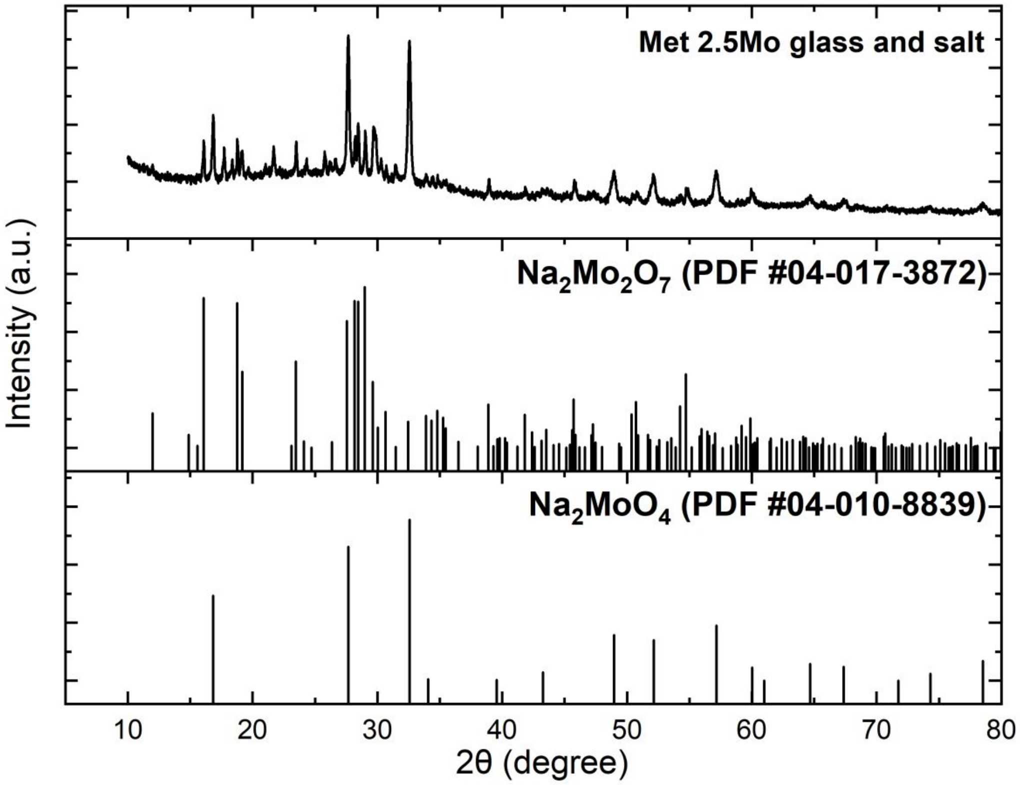

Figure S7. X-ray diffraction patterns of salt layer obtained on the surface the metaluminous glass with 2.5 mol.% MoO_3. The crystalline phases are Na_2MoO_4 (PDF #04-010-8839) and $Na_2Mo_2O_7$ (PDF #04-017-3872).

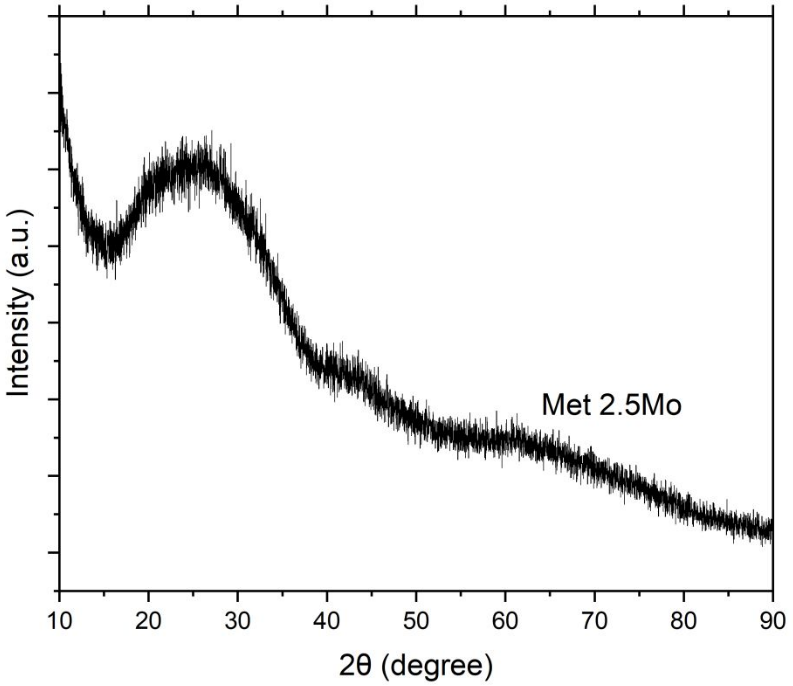

Figure S8. X-ray diffraction pattern confirming the amorphous nature of metaluminous glass containing 2.5 mol.% MoO_3.

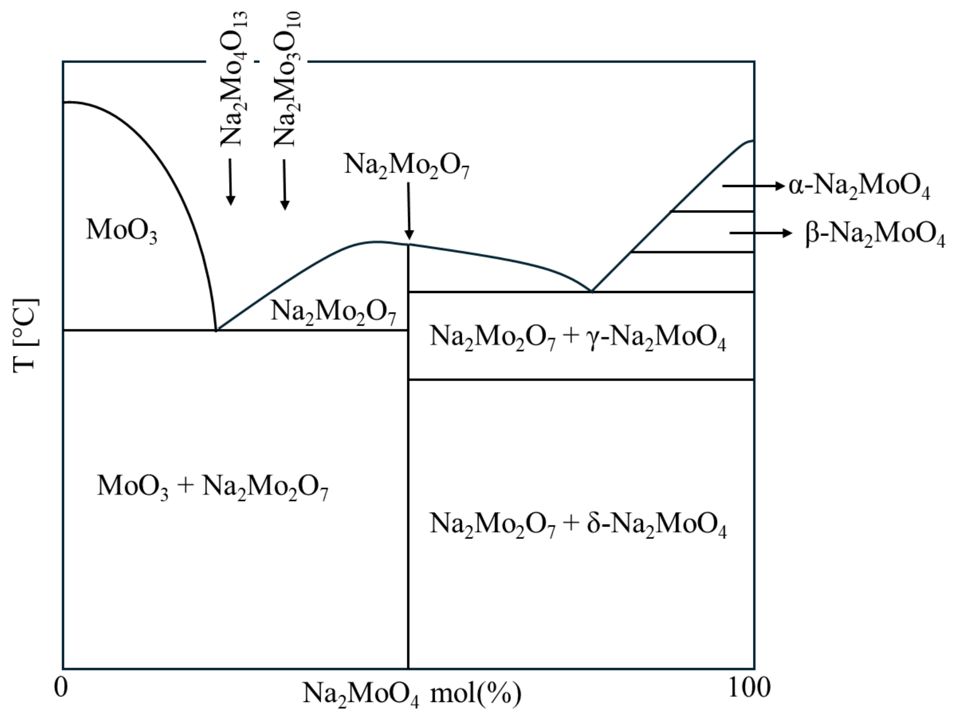

Figure S9. Reconstruction of the Na_2MoO_4-MoO_3 binary phase diagram. "Adapted with permission from [*Groschuff, E. Über Wasserfreie Molybdate I. Zeitschrift für anorganische Chemie **1908**, 58(1), 113-119*]. *Copyright [1908] [John Wiley and Sons].*"

Supporting Information

References

(1) Machida, N.; Eckert, H. FT-IR, FT-Raman and 95Mo MAS–NMR studies on the structure of ionically conducting glasses in the system AgI–Ag2O–MoO3. *Solid State Ionics* **1998**, *107* (3), 255-268.
(2) Youngman, R. NMR spectroscopy in glass science: A review of the elements. *Materials* **2018**, *11* (4), 476.
(3) Groschuff, E. Über Wasserfreie Molybdate I. *Zeitschrift für anorganische Chemie* **1908**, *58*(1), 113-119.
